# Superior Colliculus Controls the Activity of the Rostromedial Tegmental Nuclei in an Asymmetrical Manner

**DOI:** 10.1523/JNEUROSCI.1556-20.2021

**Published:** 2021-05-05

**Authors:** Kamil Pradel, Gniewosz Drwiȩga, Tomasz Błasiak

**Affiliations:** Department of Neurophysiology and Chronobiology, Institute of Zoology and Biomedical Research, Jagiellonian University, 30-387, Krakow, Poland

**Keywords:** dopamine, rostromedial tegmental nucleus, substantia nigra pars compacta, superior colliculus, ventral tegmental area

## Abstract

Dopaminergic (DA) neurons of the midbrain are involved in controlling orienting and approach of animals toward relevant external stimuli. The firing of DA neurons is regulated by many brain structures; however, the sensory input is provided predominantly by the ipsilateral superior colliculus (SC). It is suggested that SC also innervates the contralateral rostromedial tegmental nucleus (RMTg)—the main inhibitory input to DA neurons. Therefore, this study aimed to describe the physiology and anatomy of the SC–RMTg pathway. To investigate the anatomic connections within the circuit of interest, anterograde, retrograde, and transsynaptic tract-tracing studies were performed on male Sprague Dawley rats. We have observed that RMTg is monosynaptically innervated predominantly by the lateral parts of the intermediate layer of the contralateral SC. To study the physiology of this neuronal pathway, we conducted *in vivo* electrophysiological experiments combined with optogenetics; the activity of RMTg neurons was recorded using silicon probes, while either contralateral or ipsilateral SC was optogenetically stimulated. Obtained results revealed that activation of the contralateral SC excites the majority of RMTg neurons, while stimulation of the ipsilateral SC evokes similar proportions of excitatory or inhibitory responses. Consequently, single-unit recordings showed that the activation of RMTg neurons innervated by the contralateral SC, or stimulation of contralateral SC-originating axon terminals within the RMTg, inhibits midbrain DA neurons. Together, the anatomy and physiology of the discovered brain circuit suggest its involvement in the orienting and motivation-driven locomotion of animals based on the direction of external sensory stimuli.

**SIGNIFICANCE STATEMENT** Dopaminergic neurons are the target of predominantly ipsilateral, excitatory innervation originating from the superior colliculus. However, we demonstrate in our study that SC inhibits the activity of dopaminergic neurons on the contralateral side of the brain via the rostromedial tegmental nucleus. In this way, sensory information received by the animal from one hemifield could induce opposite effects on both sides of the dopaminergic system. It was shown that the side to which an animal directs its behavior is a manifestation of asymmetry in dopamine release between left and right striatum. Animals tend to move oppositely to the hemisphere with higher striatal dopamine concentration. This explains how the above-described circuit might guide the behavior of animals according to the direction of incoming sensory stimuli.

## Introduction

The role of dopaminergic (DA) neurons located in the midbrain [ventral tegmental area (VTA) and substantia nigra pars compacta (SNc)] in control of motor functions, learning, and reward-related behaviors is well established (Mogenson et al., 1980; Wise, 2004; Baik, 2013). They drive the organism to choose proper motor actions in given circumstances to maximize survival. Such action-gating property of dopaminergic neurons requires them to integrate a wide range of information, of both external and internal origin. Such a variety of information is provided by the prolific innervation from different brain areas (Watabe-Uchida et al., 2012). Sensorial information is most probably delivered by the superior colliculus (SC), which is a brain region processing sensory information from the contralateral side of the body (May, 2006; May et al., 2009; Redgrave et al., 2010). Notably, the function of intermediate and deep layers of the SC in motor guidance, driving object-oriented eye (saccades), head, body, and limb movements is well documented (Gandhi and Katnani, 2011). In contrast, superficial layers of the SC are mostly visual, involved in rapid detection of the onset of sensory stimuli and further relaying this information to the deeper SC layers.

It has been shown that SC innervates midbrain dopaminergic neurons (Comoli et al., 2003; Coizet et al., 2007; May et al., 2009; Yetnikoff et al., 2015), and the latency of the reaction of SC to visual stimuli is always shorter than that of midbrain dopaminergic neurons (Redgrave and Gurney, 2006). Phasic activity of dopaminergic neurons evoked by the visual stimuli is diminished by the lesion of the ipsilateral SC (Comoli et al., 2003). Accordingly, pharmacological disinhibition of the SC increased visually evoked (Comoli et al., 2003; Bertram et al., 2014) and spontaneous (Coizet et al., 2003) activity of midbrain dopaminergic neurons, as well as dopamine release in the striatum (Dommett et al., 2005). Moreover, when the SC of monkeys with lesioned primary visual cortex was pharmacologically blocked, it was only then that the monkeys lost the ability to perform anticipatory behaviors based on the contralateral reward-associated cues; futhermore dopaminergic neurons stopped responding to these cues (Takakuwa et al., 2017). Additionally, it was demonstrated recently that the SC–VTA pathway is important for orienting behavior during interaction with a conspecific, as well as for mediating visually evoked innate defensive responses (Prévost-Solié et al., 2019; Zhou et al., 2019).

**Figure 1.**
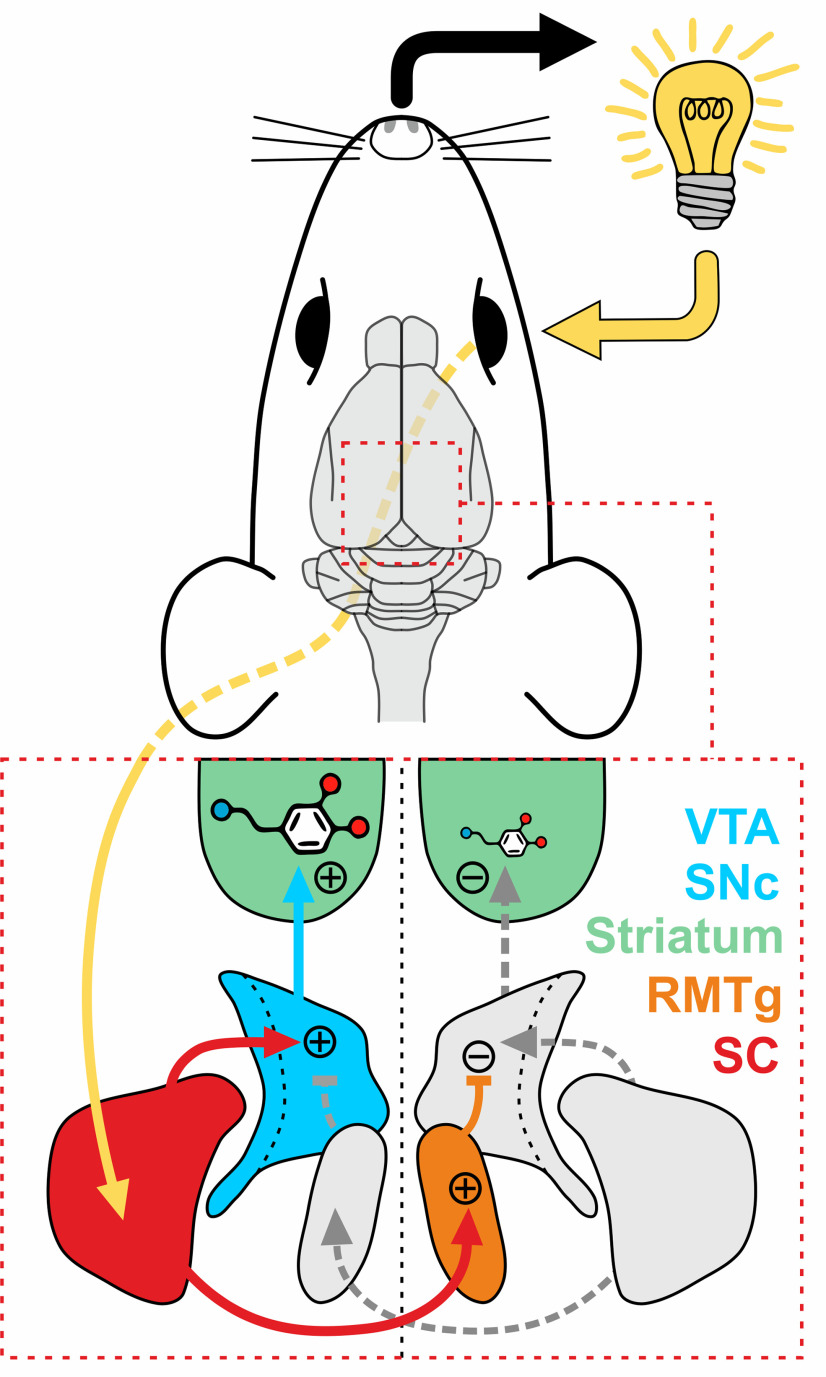
Anatomical diagram depicting the studied brain circuit. SC (red) innervates predominantly the ipsilaterally located nuclei of the midbrain dopaminergic system (VTA and SNc; blue). At the same time, SC innervates mainly the contralateral RMTg (orange). Sensory stimulus perceived on one side of the body (depicted as a light bulb) could control the activity of the dopaminergic system on both sides of the brain in an opposite manner. Such an imbalance of striatal dopamine release (depicted as a dopamine molecule) could bias the behavior of the animal toward the side from which sensory stimulus is received (rightward in the example shown; black arrow).

Studies mentioned above focus on the innervation of dopaminergic neurons descending from the ipsilateral SC. Nevertheless, anatomic studies strongly suggest that SC also sends projections to the contralaterally located rostromedial tegmental nucleus (RMTg), which constitutes the main inhibitory input to the midbrain dopaminergic neurons (Kaufling et al., 2009; Jhou et al. 2009a,b; Bourdy and Barrot, 2012; Yetnikoff et al., 2015). Given that SC innervates dopaminergic neurons located ipsilaterally and RMTg neurons located contralaterally, it can be hypothesized that the SC controls the activity of dopaminergic neurons located in both hemispheres in an opposite manner (Fig. 1).

Notably, such brain wiring might have behavioral consequences, as the side toward which the animal directs its behavior is the manifestation of the difference in the activity of the left and right dopaminergic system. Body movements and behaviors are performed toward the hemisphere with lower dopamine release and away from the hemisphere with higher dopamine release (Arbuthnott and Crow, 1971; Iwamoto et al., 1976; Joyce et al., 1981; Glick et al., 1988; Bourdy et al., 2014; Molochnikov and Cohen, 2014).

Therefore, SC, via contralateral RMTg, might contribute to the lateralization of the movement of an animal, based on the direction of incoming sensory stimuli. In this anatomic and electrophysiological study, we aimed to describe the innervation descending from the SC to the RMTg and to determine how this neuronal pathway influences the dopaminergic system. To date, this brain circuit has not been investigated.

## Materials and Methods

### 

#### 

##### Subjects.

Male Sprague Dawley rats (weight, 280–350 g) were acquired from the Institute of Zoology and Biomedical Research, Jagiellonian University (Krakow, Poland) breeding facility. Animals were housed in a room with controlled temperature (20–22°C) and humidity (40–50%) under a 12 h light/dark cycle (lights on at 8:00 A.M.). Animals had unlimited access to food and water. All procedures were performed during the light phase of the light/dark cycle. All experimental procedures were conducted according to the EU *Guide for the Care and Use of Laboratory Animals* and were approved by the Ethics Committee for Animal Experiments at the Institute of Pharmacology, Polish Academy of Sciences (Krakow, Poland).

##### Brain injection surgeries.

All the procedures were conducted under deep anesthesia induced by intraperitoneal injection of ketamine and xylazine (100 and 10 mg/kg body mass, respectively; Biowet-Puławy). The reflexes of the animals were checked to assure that the anesthesia was sufficient to conduct the surgical procedure. During the surgery, the deep body temperature of animals was controlled and held at 37°C by an automatic heating pad (temperature controller TCP-02, WMT). Animals were carefully placed in a stereotaxic frame (model SF-4100, ASI Instruments), using standard noninvasive ear bars (45° tip; model EB-945), and an incisor bar (model RA-200). A sagittal incision on the top of the head was made and the skin and soft tissue covering the bones were retracted to expose the sutures. Once the bregma and lambda points were evenly positioned in the dorsoventral axis, small craniotomies were performed to allow the intrabrain injections. For injections, Hamilton syringes (0.5 or 1 µl; Hamilton) connected to glass micropipettes (20–40 µm tip) via 3-cm-long Tygon tubing were used. Micropipettes were prepared from glass capillaries (Vitrex Medical A/S) using a vertical puller (model PE-21, Narishige International Instruments), and the whole injection system was filled with paraffin oil (Sigma-Aldrich). Stereotaxic coordinates used were obtained from a rat brain atlas (Paxinos and Watson, 2007) prior to experiments and were refined experimentally. For retrograde tract-tracing experiments, 40 nl of FluoroGreen (Tombow Pencil) was injected unilaterally into the RMTg using the following stereotaxic coordinates: 10° lateral angle, −6.8 to −7.0 mm caudally, +1.7 mm laterally, and −7.4 mm ventrally from the skull surface at the bregma point. For anterograde tracing experiments, 400 nl of AAV2-hSyn-eYFP [enhanced yellow fluorescent protein; viral titer: 5.2 × 10^12^ viral genomes (vg)/ml; UNC Vector Core, University of North Carolina, Chapel Hill, NC] was injected into the superior colliculus using the following coordinates: −6.4 mm caudally, +2.2 mm laterally, and −4.4 mm ventrally from the bregma point. For electrophysiological experiments, injections of 300–400 nl of AAV2-hSyn-ChR2(H134R)-eYFP (viral titer: 3.1 × 10^12^ vg/ml; UNC Vector Core) into the SC (unilaterally or bilaterally) were performed using the following coordinates: −6.4 to −6.6 mm caudally, ±1.7 to ±2.2 mm laterally, and −4.4 to −4.6 mm ventrally from the bregma point. In the transsynaptic tract-tracing experiments, 80–200 nl of AAV1-hSyn-Cre-hGH (AAV; viral titer: 1.8 × 10^13^ vg/ml; Addgene) was injected into the SC unilaterally (−6.4 to −6.6 mm caudally, ±1.8 to ±2.0 mm laterally, and −4.4 to −4.6 mm ventrally from the bregma point), and, in some of these rats, 300 nl of AAV2-EF1α-DIO-mCherry (viral titer: 5.3 × 10^12^ vg/ml; UNC Vector Core) was injected into the contralateral RMTg (10° lateral angle, −6.8 to −7.0 mm caudally, +1.7 mm laterally, and −7.4 mm ventrally from the bregma point). In the transsynaptic electrophysiological experiments, the procedure was the same, with one difference: AAV2-EF1α-DIO-ChR2(H134R)-mCherry (viral titer: 5.1 × 10^12^ vg/ml; UNC Vector Core) was used. Injections were performed at the rate of 100 nl/min, and once they were done the pipette was held in place for at least 5 min before retracting. Finally, the skin incision was sutured and antibacterial balm (Tribiotic; Kato Labs) was placed on the wound. Afterward, the animals were subcutaneously injected with the anti-inflammatory drug Tolfedine (4 mg/kg body mass; Biowet-Puławy) and the painkiller Torbugesic (0.2 mg/kg body mass; Biowet-Puławy) and then placed in their home cage to recover. For 5 d after the surgery, the animals were treated with antibiotics added to their drinking water (Sul-Tridin 24%, 1 ml/300 ml water; Biowet-Puławy).

##### Anatomical experiments.

For the purpose of anatomic experiments, animals were killed at the following times: 1 week after the FluoroGreen injection in the case of retrograde tracing; 6 weeks after viral injections in the case of anterograde tracing; and 3 weeks after viral injections in the case of transsynaptic tracing (details of surgery are described in the previous section). Animals were transcardially perfused with 300 ml of PBS, pH ∼7.4, followed by 300 ml of 4% formaldehyde in PBS, and, after extraction, their brains were submerged in such a fixing solution for another 24 h. Afterward, using a vibratome (model VT1000S, Leica) the brains were cut in the coronal plane into 50-µm-thick slices. The slices were then mounted on a glass slide with the use of a DAPI-containing mounting medium (VectaShield, Sigma-Aldrich), covered with a coverslip, and then investigated under a fluorescent microscope [Axio Imager.M2 (equipped with an AxioCam MRm camera), Zeiss]. Once assured that tracers were properly injected into the desired brain areas, coronal slices from different distances from the bregma in the anteroposterior (AP) axis were photographed to capture images of the entire areas of the brain that were investigated. Superior colliculi (retrograde tracing) were photographed at −5.6, −6.0, −6.5, −6.9, and −7.3 mm from the bregma, in panorama mode, under 10× magnification. RMTg slices (anterograde tracing) were photographed at −6.4, −6.9, and −7.4 mm from the bregma, in *z*-stack mode (3-µm-depth spacing), under 20× magnification. In cases of transsynaptic anterograde tracing, in rats with two viral injections (for details, see Brain injection surgeries), representative brain slices for the SC and RMTg, as well as five to six slices of the VTA/SNc between −5 and −5.9 mm from the bregma, were photographed under 10× magnification in the panorama mode. In rats with a single injection (for details, Brain injection surgeries), representative brain slices for the SC and three slices of the RMTg at −6.6, −7.1, and −7.6 mm from bregma were photographed.

In the case of transsynaptic anterograde tracing experiments, before microscope inspection, brain slicing was followed by the immunohistochemical staining procedure. First, blocking of the nonspecific binding sites and membrane permeabilization was performed for 1 h at room temperature [10% normal donkey serum (NDS) and 0.3% Triton X-100 diluted in PBS; Jackson ImmunoResearch Europe and Sigma-Aldrich, respectively]. Next, slices containing injection sites and, in the case of brains with single injection, slices of RMTg were incubated for 2 d in 4°C temperature with a primary antibody against Cre-recombinase (murine anti-Cre antibody 1:1000, 2% NDS, 0.3% Triton X-100 diluted in PBS; Abcam). In cases of VTA and SNc slices, immunostaining against tyrosine hydroxylase was performed [rabbit anti- tyrosine hydroxylase (TH) antibody 1:500, 2% NDS, 0.3% Triton X-100 diluted in PBS; Sigma-Aldrich]. Then, slices were washed three times with PBS for 10 min and then incubated for the next 24 h in 4°C temperature with a secondary antibody (donkey anti-mouse antibody with Alexa Fluor 647 1:400 or donkey anti-rabbit antibody with Alexa Fluor 488 1:400, 2% NDS diluted in PBS; Jackson ImmunoResearch Europe).

Since RMTg is a brain region whose anatomic boundaries are difficult to determine from landmarks in unstained tissue, we performed immunostaining against FoxP1, as it has recently been shown to serve as a robust RMTg marker (Smith et al., 2019). For this purpose, blocking and membrane permeabilization were performed as described above in brain slices collected from naive animals. Then, RMTg slices were incubated for 1 d in 4°C temperature with a primary antibody against FoxP1 (rabbit anti-FoxP1 antibody 1:5000, 2% NDS, 0.3% Triton X-100 diluted in PBS; Abcam). Next, the slices were washed three times with PBS and incubated with the secondary antibody (donkey anti-rabbit antibody with Cy3 1:400, 2% NDS diluted in PBS; Jackson ImmunoResearch Europe) for the next 24 h in 4°C temperature. Based on the staining obtained, a map was prepared and used to determine the limits of RMTg in this study.

##### Electrophysiological experiments.

Electrophysiological experiments were performed at least 2 weeks after the viral vector injections (for details, see Brain injection surgeries). Animals were deeply anesthetized by intraperitoneal injection of urethane (1.5 g/kg body mass; Sigma-Aldrich) diluted in 0.9% sodium chloride. During the procedure, the deep body temperature of the animals was controlled and held at 37°C by an automatic heating pad (temperature controller TCP-02, WMT) via rectal thermometer. Animals were then mounted to a stereotaxic frame (model SF-1450AP, ASI Instruments) using ear bars (18° tip; model EB-918) and incisor bar (model RA-200). A sagittal incision on the head was performed, and the skin and soft tissue covering the bones were retracted to expose the sutures. After ensuring that bregma and lambda points were evenly positioned in the dorsoventral axis, craniotomies were made to allow the implantation of electrodes and optical fibers. All exposed brain surfaces were covered with paraffin oil (Sigma-Aldrich) to prevent tissue from drying.

Extracellular recordings of RMTg neuronal activity were conducted using 32-channel silicon probes (four shanks; 200 µm shank spacing; 50 µm recording spot spacing; 1–2 MΩ impedance; model A4X8-10 mm-50–200-177, NeuroNexus). The extracellular signal was digitized (40 kHz/channel), wide-band filtered (0.77–7500 Hz), and stored on a hard drive using the OmniPlex D Neural Recording Data Acquisition System (Plexon). Shanks of the electrode were placed in the RMTg using the following stereotaxic coordinates: 10° lateral angle, −7.8 to −6.8 mm caudally, −2.3 to −2.5 mm laterally, and −7.2 to −8.8 mm ventrally from the bregma. Recordings from three to five positions, with the spacing of 0.4 mm in the dorsoventral axis, were performed. To place the electrode within the anterior part of the RMTg while leaving the SC intact, the skull was positioned at an ∼8° angle by lowering the bregma below the level of lambda, and then the following coordinates were used for the electrode positioning: −8.1 to −8.7 mm caudally, −2.3 mm laterally, and −6.6 to −7.8 mm ventrally from the bregma. In this configuration, recording from three positions, with the spacing of 0.4 mm in the dorsoventral axis, was performed. To reduce tissue damage, a hydraulic micromanipulator was used to place MEA (multielectrode array) in the desired position (MO-10, Narishige). To aid probe placement verification, it was covered with fluorescent dye before brain implantation (DiI, DiO, or DiD dye; Thermo Fisher Scientific).

Extracellular single-cell recordings in VTA/SNc were performed using micropipettes prepared from borosilicate glass capillaries (outer diameter, 1.5 mm; inner diameter, 0.86 mm, with filament; Sutter Instrument) using a horizontal puller (model P-97, Sutter Instrument). Glass electrodes were filled with 2% Chicago Sky Blue dye diluted in 0.5 m NaCl. Electrodes with an impedance between 20 and 35 MΩ were used for the recordings. Such electrodes were placed within the VTA or SNc using the following stereotaxic coordinates: −5 to −6.0 mm caudally, 0.8-1.7 mm laterally, and −7.8 to −9 mm ventrally from the skull surface at the bregma point. To increase the stability of the recording, a hydraulic micromanipulator was used to place the electrode in the desired position (model MO-10, Narishige). Signals were amplified and filtered (1000×, 300–5000 Hz) using a BA-03x bridge amplifier (NPI Electronic). Analog signal was digitized (40 kHz) with a Micro1401 mk II interface operated by Spike2 software for recording, storage, and further analysis (Cambridge Electronic Design). Once the recordings were made, a negative current (−10 μA) was passed through the electrode for 15–20 min to deposit Chicago Sky Blue dye at the recording site (Stimulus Isolator, WMT). Recorded neurons were classified as dopamine-like when they met previously established electrophysiological criteria: a triphasic broad action potential (>1.1 ms measured from the action potential initiation to the minimum of the trough) and a firing rate <10 Hz (Grace and Bunney, 1980, 1983; Ungless and Grace, 2012). The final criterion for classifying a neuron meeting the electrophysiological criteria as a DA-like neuron was the histologic confirmation of its position in the VTA/SNc.

For optogenetic stimulation of the SC during RMTg recordings, the tips of two optical fibers (Ø, 105 µm; numerical aperture, 0.22; ThorLabs), each connected to a 473 nm laser source (MBL-III-473 laser with PSU-III-LED controller; CNI Optoelectronics Technology Co, Ltd.), were placed just above the left and right SC using the following stereotaxic coordinates: −6.6 to −7.0 mm caudally, ±1.7 to ±2 mm laterally, and −3 to −3.5 mm ventrally from the bregma. When the skull was positioned at the ∼8° angle, the following coordinates were used: 8° anterior angle, −7 mm caudally, ± 1.7 to ±1.9 mm laterally, and −2.4 to −2.6 mm ventrally from the bregma. In the case of VTA/SNc recordings, to stimulate RMTg neurons innervated by the contralateral SC or SC-originating axon terminals in the contralateral RMTg, optical fiber was placed just above the RMTg using the following stereotaxic coordinates: −7.0 to −7.8 mm caudally, 1.9-2 mm laterally, and −7 mm ventrally from the bregma. To aid optical fiber placement verification, they were covered with fluorescent dye before brain implantation (DiI, Thermo Fisher Scientific). The power of the blue light emitted from the tip of the optical fibers was measured by a photodiode power sensor (model S121C, ThorLabs) connected to a digital optical power and energy meter (model PM100D, ThorLabs). The analog signal provided by the light meter was further digitalized by Micro1401 mk II interface and accurately measured in Spike2 software (Cambridge Electronic Design) to check the stability of the light source. Each light stimulation was delivered into the brain tissue with a power of under 20 mW at the fiber tip, measured before each experiment. For the RMTg experiments, single 100 ms light pulses were used, repeated every 6 s. For the VTA/SNc experiments, single 100 ms light pulses (repeated every 6 s) or 5 ms pulses delivered at 40 Hz for 1 s (repeated every 10 s) were used.

Custom-made GUI and scripts in Spike2 were used to apply the light protocols for the purpose of optogenetic stimulation. Additionally, electrocorticographic (ECoG) recordings were conducted using a silver wire connected to a screw (0.1–0.2 MΩ measured at 1 kHz in saline) that was placed over the right hemisphere at the border of the primary motor and somatosensory cortices. The ECoG was performed to avoid the comparison between the effects of the contralateral and ipsilateral stimulation of the SC observed in the different states of the brain (i.e., activation and slow-wave activity), as the activity of neurons might be altered by the brain state. At the end of each experiment, the animals were injected with 0.5 ml of pentobarbital (Morbital, Biowet-Puławy) and killed by transcardial perfusion with PBS, pH ∼7.4, followed by 4% formaldehyde in PBS. The brains were extracted and held in 4% formaldehyde in PBS for at least 24 h. Then the brains were sliced and inspected under the fluorescent microscope, as described above in the Anatomical experiments section. Slices containing the tip of silicon probe (DiI, DiO, or DiD dye) or Chicago Sky Blue dye deposition, the tips of optical fibers (DiI dye), as well as eYFP- or Cre-expressing SC neurons or mCherry-expressing RMTg neurons, were photographed. The images were adjusted to the corresponding section of the stereotaxic atlas of the rat brain (Paxinos and Watson, 2007) using CorelDRAW software (Corel) to verify both the placement of each recording spot as well as the optogenetic stimulation site. In cases of transsynaptic VTA/SNc electrophysiological experiments, slices containing SC were immunohistochemically stained against Cre-recombinase (details are described above in the Anatomical experiments section). Only the recordings that were localized within the borders of RMTg or VTA/SNc—and only those from brains where proper eYFP/Cre/mCherry expression and optical fiber placement was observed—were further analyzed.

##### Data analysis.

Anatomical data were analyzed using ImageJ software (FIJI, version 1.52s). Panoramic images of both superior colliculi, after retrograde tracing of the SC–RMTg neuronal pathway, were inspected using the Cell Counter plugin. Neuronal tracer-filled neurons were manually marked in each layer of the superior colliculi on both contralateral and ipsilateral sides in relation to the RMTg injection site. The position of each neuron was extracted and used for further analysis of the spatial distribution using custom-made MATLAB scripts and for statistical comparisons. In cases of anterograde tracing of the SC–RMTg neuronal pathway, the *z*-stack images of both RMTgs obtained from brains unilaterally injected with anterograde tracer into the SC (for details, see Brain injection surgeries) were analyzed. The area fraction taken by the eYFP-positive axon terminals present in both RMTgs was calculated by first changing the photograph to an 8 bit image, then subtracting the background (rolling ball radius = 50 pixels) and projecting the *z*-stack onto one plane (projection type: max intensity). Then, the image was sharpened (Unsharp Mask: radius = 10, mask weight = 0.9), denoised (three times, Despeckle), binarized (threshold range: 45–255 pixels), small artifacts were removed (Analyze Particles: size = 80 to infinity pixels), and, finally, the area fraction was calculated. In the case of anterograde transsynaptic tracing, Cre-positive neurons within the RMTg were manually marked on both contralateral and ipsilateral sides in relation to the SC injection site (in rats with AAV1-Syn-Cre-hGH-only injection). Then, the positions of these neurons were extracted and used for further visualization of spatial distribution using custom-made MATLAB scripts and for statistical comparisons. In rats with the following injection of the Cre-dependent AAV, within the ventral midbrain the density of axons originating in the RMTg neurons innervated by the contralateral SC were measured. For that purpose, VTA and SNc were outlined (based on anti-TH staining). Next, the photograph was changed to an 8 bit image, the background was subtracted (rolling ball radius = 50 pixels) and the image was sharpened (Unsharp Mask: radius = 5, mask weight = 0.3–0.9), denoised (three times, Despeckle), binarized (threshold range: 30–255 pixels), and the region outside the VTA and SNc was filled with gray color. Such images were subjected to further analysis using a custom-made MATLAB script where the area fraction was calculated for each 0.2 mm bin in the mediolateral axis and further visualized. For each brain, five to six slices were averaged.

To analyze the electrophysiological data obtained during RMTg recordings, the recorded .pl2 files (Plexon file format) were converted to binary format, and spikes were detected and sorted using Kilosort (KilosortPLX) software (Pachitariu et al., 2016) in MATLAB environment (version R2018a). The GPU (graphics processing unit; NVIDIA GeForce GTX 1060 Max-Q; CUDA version 9.0 for Windows) was used for all of the calculations. Using the custom-made MATLAB scripts, the results of spike sorting were transferred to .smrx files (Spike2 file format) containing a bandpass-filtered signal (300–7500 Hz bandpass filter, Butterworth, fourth order). The signal from each recording spot that was previously found to be located in the RMTg was then manually inspected. If needed, the signal was further refined (cleaned, split, merged) by using custom-made Spike2 scripts. Autocorrelograms, PCA, as well as a visual comparison of the separated unit and raw signal, were used as indicators of proper separation of spikes from the raw signal. Once all the units were inspected and refined, a custom-made Spike2 script was used to calculate the average peristimulus spike density function (SDF; Gaussian kernel, width = 100 ms) and saved to .txt files. The same script and parameters were used to calculate peristimulus spike density function from single channel recordings in the VTA/SNc (from files that were inspected, cleaned, and refined in advance). Next, custom-made MATLAB scripts were used for preparing the heatmaps and the mean and median activity plots and for the detection of the effects caused by SC optogenetic stimulation (excitation or inhibition). Part of the data is presented as normalized firing rate, which was calculated by dividing all the bins by the mean value of all bins from the baseline, so the mean baseline is equal to 1. The neuronal activity was considered to be altered (either excited or inhibited) by optogenetic stimulation when SDF crossed the threshold of 1 SD above (or below) the mean value of SDF in the baseline, and additionally, the length of such change had to be longer than the mean length (plus 1 SD) of threshold-crossing episodes in the baseline. Threshold crossing appearing later than 60 ms after the onset of the optogenetic stimulation was not considered as an effect. In the case of experiments where axon terminals from SC were stimulated within the borders of the contralateral RMTg, the neuron was considered as excited (or inhibited) when its activity during the stimulation period (1 s) was higher (or lower) than the average activity in baseline ± SD. The obtained data underwent statistical analysis (see below).

##### Experimental design and statistical analysis.

All statistical analyses were performed using GraphPad Prism software (version 6.0 for Windows; GraphPad Software) or SPSS Statistica (version 13 for Windows; Tibco Software). The complete results of the statistical analyses were reported in the Results section. All data are presented as the mean ± SEM, unless otherwise stated. Compared values were considered to be significantly different at *p* < 0.05. The distribution of the data was tested (Kolmogorov–Smirnov and Shapiro–Wilk tests) before choosing the appropriate test for the analysis. In the case of normally distributed data, the Student's *t* test or one-way ANOVA was used (paired, if possible). In the case of data that were not normally distributed, the Mann–Whitney test (not paired), Wilcoxon matched-pairs signed-rank test (paired), Kruskal–Wallis test (not paired), or Friedman test (paired) was used. If more than one factor was present, a two-way ANOVA or three-way ANOVA was used. When more than two groups were compared, the tests were followed by Dunnett's or Tukey's *post hoc* test (parametric) or Dunn's *post hoc* multiple-comparison test (nonparametric). Multivariate ANOVA (MANOVA) test was used if more than one dependent variable was tested simultaneously. In the case of contingency tables, the χ^2^ test was used. For the correlations, the Kendall's τ rank correlation coefficient test was used, since the data were not normally distributed and the sample size was small.

### Data availability

The custom-made MATLAB and Spike2 codes that were used for analyses are available from the corresponding authors on individual request.

## Results

### Anatomical lateralization of neuronal pathway descending from SC to RMTg

**Figure 2.**
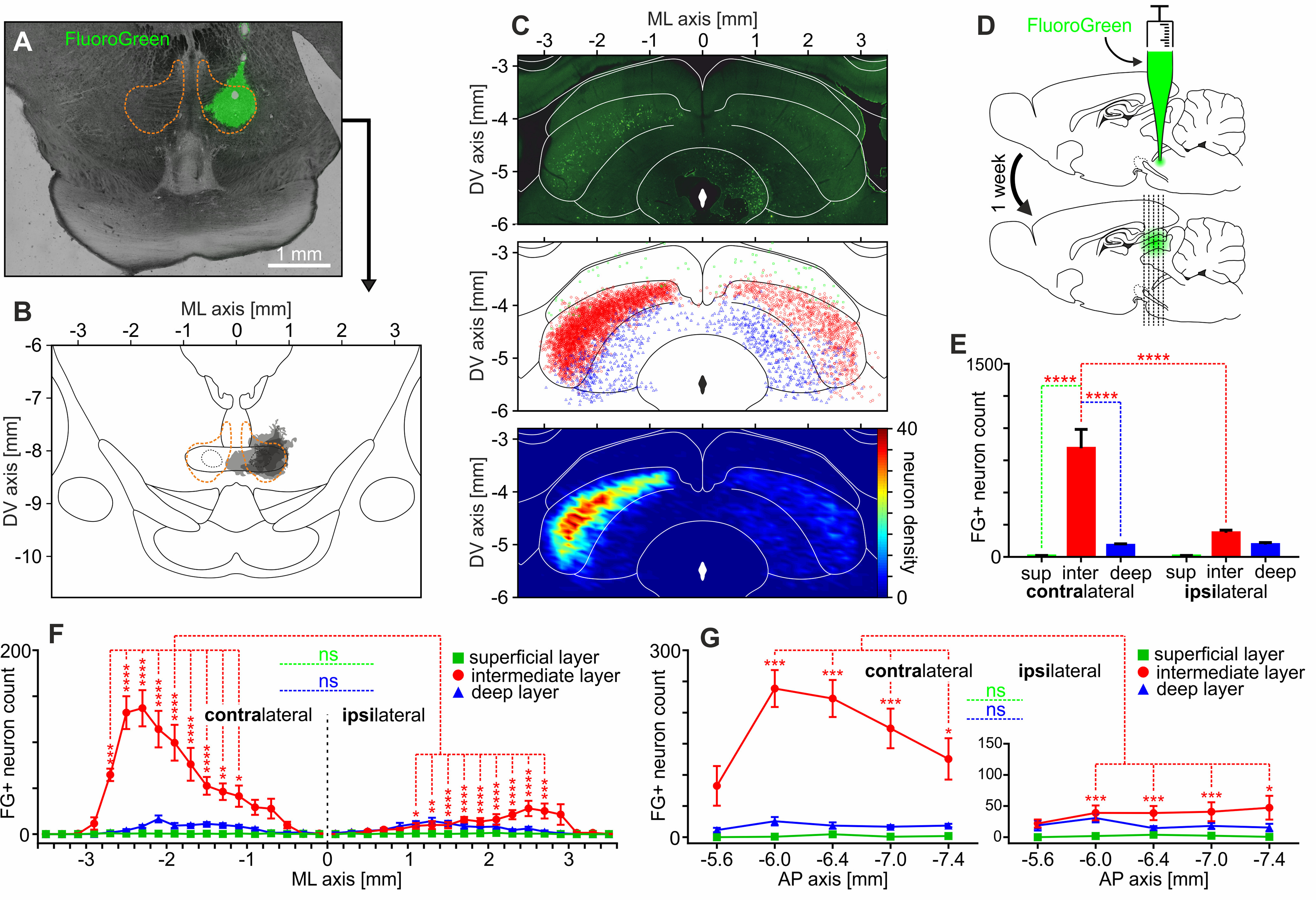
RMTg is innervated predominantly by the lateral parts of the intermediate layer of the contralateral SC. ***A***, Exemplary image of FluoroGreen injection site within the RMTg. The orange dashed line indicates the RMTg boundaries based on the anti-FoxP1 immunostaining. ***B***, Reconstruction of the FluoroGreen spread at the sites of injections performed in all rats (*n* = 5). Darker color indicates the overlap of injection across rats. The orange dashed line indicates the RMTg boundaries based on the anti-FoxP1 immunostaining. ***C***, Top, Exemplary image showing retrogradely filled SC neurons after the unilateral injection of FluoroGreen into the RMTg. Middle, Reconstruction of position of retrogradely filled SC neurons observed in all rats (*n* = 5). Green squares, superficial layers; red circles, intermediate layers; blue triangles, deep layers. Bottom, Density plot showing all retrogradely filled SC neurons. ***D***, Scheme of the experiment. RMTg was unilaterally injected with FluoroGreen, and after 1 week SC was inspected in search of retrogradely labeled neurons. ***E***, Average count of FluoroGreen-positive neurons in both contralateral and ipsilateral SC with regard to SC layers and laterality. ***F***, Distribution of FluoroGreen-positive neurons in mediolateral axis with regard to both SC layers and laterality. ***G***, Distribution of FluoroGreen-positive neurons in anteroposterior axis with regard to both SC layers and laterality. *p < 0.05, **p < 0.01, ***p < 0.001, ****p < 0.0001. ns, Nonsignificant.

To precisely describe the neuronal pathway descending from SC to RMTg, different tract-tracing techniques were used. The superior colliculi of five rats, which received unilateral injection of the neuronal tracer (FluroGreen) into the RMTg, were inspected in search of retrogradely labeled cells (Fig. 2*A*,*B*,*D*). The vast majority of FluoroGreen-positive neurons were found contralaterally to the injection site, as depicted in the exemplary SC photography (Fig. 2*C*, top), the aggregate depiction of all neurons found (Fig. 2*C*, medium), and density plot of all the neurons (Fig. 2*C*, bottom). The highest number of retrogradely labeled cells were located in the intermediate layer of SC contralateral to the injection site (Fig. 2*E*; *n* = 5, two-way ANOVA; overall contralateral and ipsilateral cell count: 942.6 ± 157.8 and 293.4 ± 25.4; laterality: *F*_(1,24)_ = 18.96, *p* = 0.0002; layer: *F*_(2,24)_ = 39.84, *p* < 0.0001; laterality × layer interaction: *F*_(2,24)_ = 19.58, *p* < 0.0001; followed by Tukey's *post hoc* test—contralateral intermediate vs ipsilateral intermediate: 843.8 ± 146.3 vs 187.6 ± 20.88, *q* = 10.8, *p* < 0.0001; contralateral intermediate vs contralateral superficial: 843.8 ± 146.3 vs 7.8 ± 4.07, *q* = 13.74, *p* < 0.0001; contralateral intermediate vs contralateral deep: 843.8 ± 146.3 vs 91 ± 11.36, *q* = 12.37, *p* < 0.0001; other comparisons did not reveal significant differences). This demonstrates that RMTg is predominantly innervated by the intermediate layer of the contralateral SC. Notably, the number of labeled neurons in the intermediate layer of the SC increased with laterality (Fig. 2*F*). The difference in FluoroGreen-positive neuron count between the contralateral and ipsilateral sides was marked only in the intermediate layer of the SC, at the lateralities between 1.1 and 2.7 mm (Fig. 2*F*; *n* = 5, three-way ANOVA; laterality: *F*_(1,432)_ = 145.07, *p* < 0.0001; layer: *F*_(2,432)_ = 304.84, *p* < 0.0001; ML: *F*_(17,432)_ = 22.91, *p* < 0.0001; laterality × layer interaction: *F*_(2,432)_ = 149.81, *p* < 0.0001; laterality × ML interaction: *F*_(17,432)_ = 10.93, *p* < 0.0001; ML × layer interaction: *F*_(34,432)_ = 17.19, *p* < 0.0001; laterality × layer × ML interaction: *F*_(34,432)_ = 9.90, *p* < 0.0001; followed by *post hoc* Tukey's test). Similarly, in the case of the anteroposterior axis, the difference in FluoroGreen-positive cell count between contralateral and ipsilateral sides was seen only in the intermediate layer of the SC throughout the whole SC, except the most anterior part (Fig. 2*G*; *n* = 5, three-way ANOVA: laterality: *F*_(1,120)_ = 69.07, *p* < 0.0001; layer: *F*_(2,120)_ = 145.12, *p* < 0.0001; AP: *F*_(4,120)_ = 5.17, *p* = 0.0007; laterality × layer interaction: *F*_(2,120)_ = 71.33, *p* < 0.0001; laterality × AP interaction: *F*_(4,120)_ = 3.20, *p* = 0.0154; AP × layer interaction: *F*_(8,120)_ = 3.61, *p* = 0.0008; laterality × layer × AP interaction: *F*_(8,120)_ = 3.13, *p* = 0.003; followed by *post hoc* Tukey's test). Also, it was observed that the most SC neurons innervating contralateral RMTg were located in the central part of the SC in the AP axis (Fig. 2*G*). To summarize, the results of retrograde tract tracing revealed that RMTg is innervated predominantly by the lateral parts of the intermediate layer of the contralateral SC.

**Figure 3.**
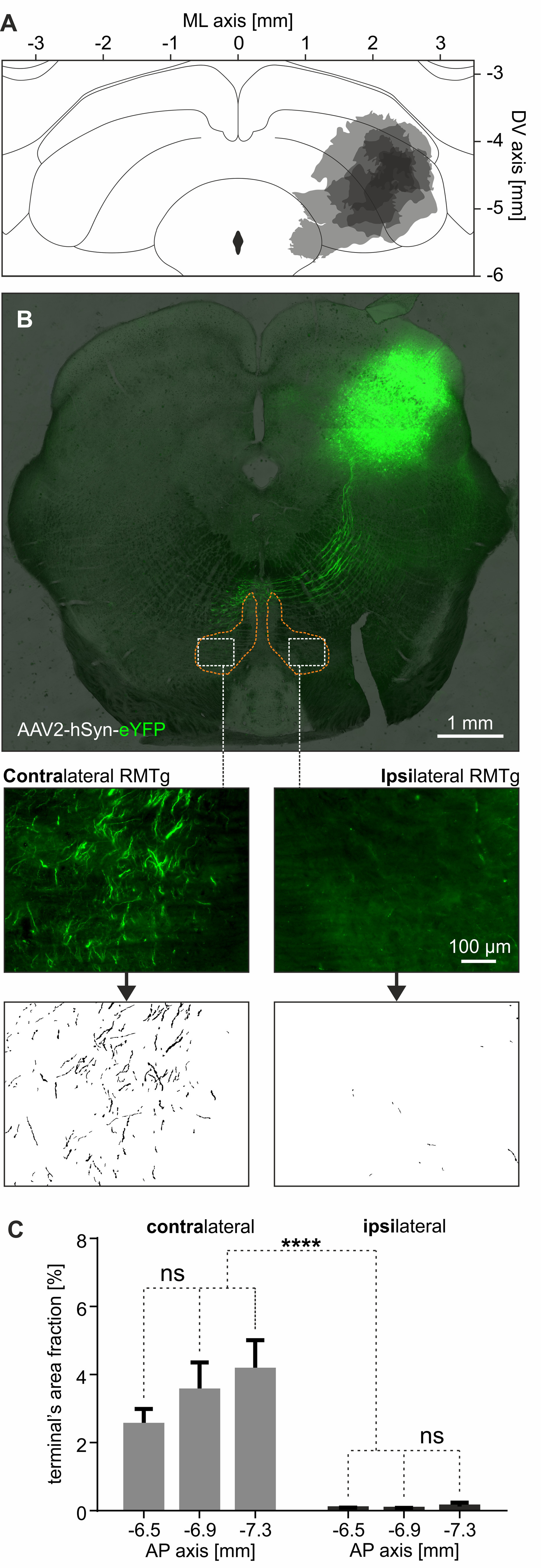
Axon terminals from SC are present within the borders of contralateral RMTg. ***A***, Reconstruction of injection sites of AAV2 containing gene for eYFP into the SC of all five rats. Darker color indicates the overlap of injections across rats. ***B***, Top, Exemplary image of the brain of the rat showing eYFP-expressing axons descending from SC. The orange dashed line indicates the RMTg boundaries based on the anti-FoxP1 immunostaining. Middle, Example of images taken from the anterior RMTg part on both contralateral (left) and ipsilateral (right) side where SC-originating axons are visible. Bottom, Example of binarized axons within the RMTg. ***C***, Quantification of binarized SC-originating axons in both contralateral and ipsilateral RMTg at different AP levels. ****p < 0.0001. ns, Nonsignificant.

The above observations were further confirmed by the results of anterograde tract-tracing experiments. The rostromedial tegmental nuclei of five rats that received a unilateral injection of AAV2-hSyn-eYFP into the SC (Fig. 3*A*) were investigated for the presence of SC descending axon fibers (Fig. 3*B*). The area fraction of SC axons within the contralateral RMTg was significantly higher than in the ipsilateral RMTg; however, no difference was seen within the anteroposterior axis (Fig. 3*B*,*C*; *n* = 5, two-way ANOVA; laterality: contralateral, 3.40 ± 0.47%; vs ipsilateral, 0.08 ± 0.04%; *F*_(1, 24)_ = 58.93, *p* < 0.0001; AP: *F*_(2,24)_ = 1.27, *p* = 0.30; laterality × AP interaction: *F*_(2,24)_ = 1.12, *p* = 0.34). This clearly shows that axons descending from SC are present densely in the contralateral RMTg while being almost absent in the ipsilateral RMTg.

**Figure 4.**
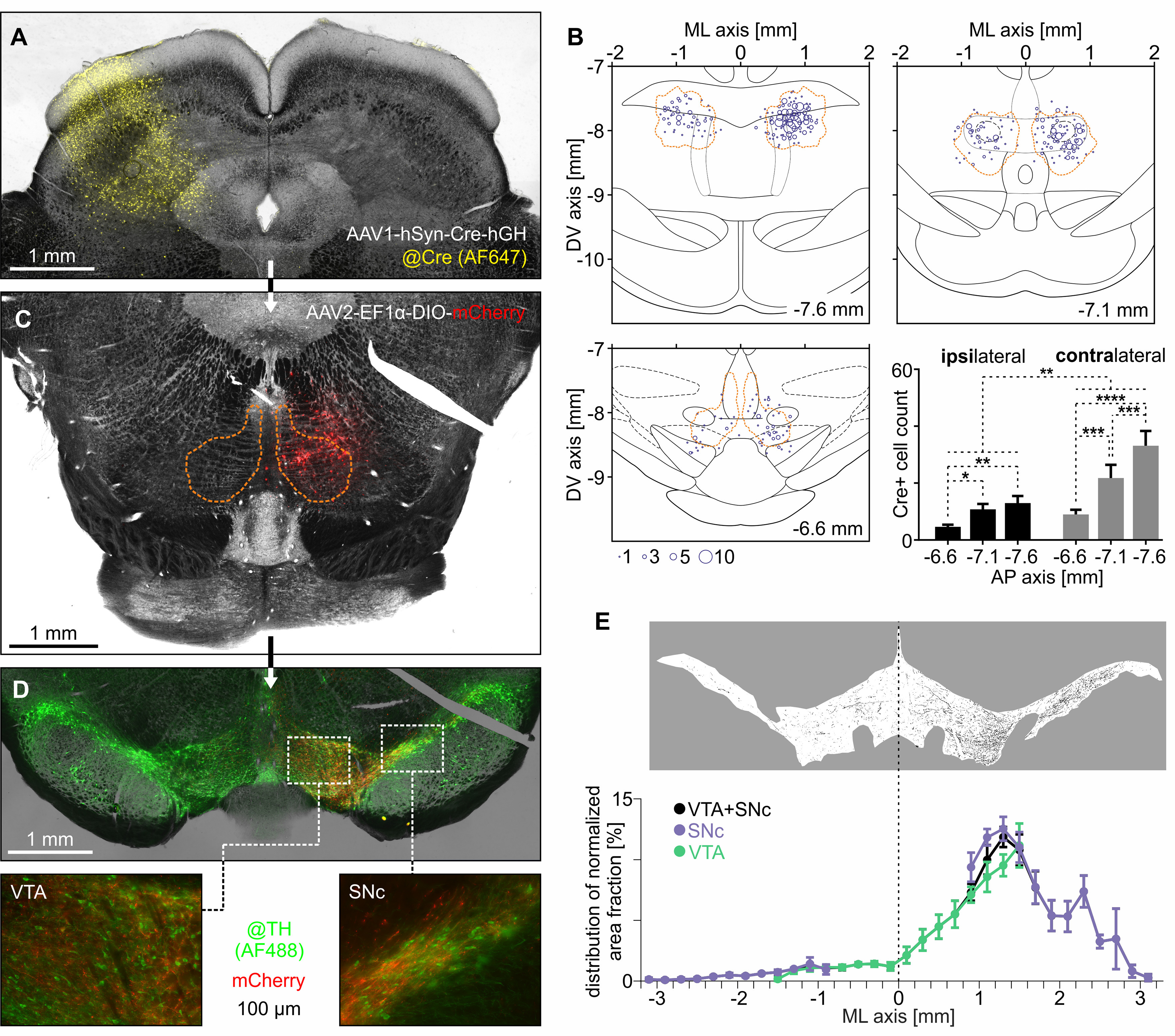
RMTg neurons monosynaptically innervated by the contralateral SC project to the midbrain dopaminergic system. ***A***, Exemplary brain image showing the site of unilateral injection of anterograde transsynaptic AAV (AAV1-hSyn-Cre-hGH) containing the gene for Cre recombinase into the SC. Immunohistochemical staining was performed to visualize Cre-recombinase (Alexa Fluor 647; yellow color). ***B***, Transsynaptically labeled Cre-positive neurons within RMTg. The orange dashed line indicates the RMTg boundaries based on the anti-FoxP1 immunostaining. ***C***, Representative image of RMTg after the follow-up injection of AAV (AAV2-EF1α-DIO-mCherry) carrying Cre-dependent gene for fluorescent protein (mCherry; red color). The orange dashed line indicates the RMTg boundaries based on the anti-FoxP1 immunostaining. ***D***, Top, Representative image of VTA and SNc after the injections of anterograde transsynaptic AAV (containing Cre recombinase gene) into the SC and AAV with Cre-dependent gene for mCherry into the contralateral (right in this case) RMTg. Immunohistochemical staining was performed to visualize TH-positive neurons (Alexa Fluor 488; green color). Bottom panels, Magnified regions of VTA and SNc with mCherry-expressing axons originating in the RMTg neurons innervated by the contralateral SC. ***E***, Top, Exemplary image of binarized mCherry-positive axons within the VTA and SNc used for area fraction calculation. Bottom, Mediolateral distribution of normalized area fraction of mCherry-expressing axons originating from the RMTg neurons innervated by the contralateral SC. *p < 0.05, **p < 0.01, ***p < 0.001, ****p < 0.0001.

These results were further corroborated by the transsynaptic tracing experiments. Nine animals received injections of AAV1-Syn-Cre-hGH into the SC and three of them were also injected with AAV2-EF1α-DIO-mCherry into the contralateral RMTg. The brain slices containing SC, RMTg, VTA, and SNc underwent immunohistochemical staining procedures against either Cre-recombinase or tyrosine hydroxylase. Then the slices were investigated with the use of fluorescent microscopy. Transduced SC neurons transport the viral vector anterogradely through the axon and pass one synapse, causing Cre expression in monosynaptically innervated neurons (Zingg et al., 2017; Huang et al., 2019). The expression of Cre-recombinase at the injection site can be seen in Figure 4*A*. Therefore, the mCherry expression from the second viral vector relied on the monosynaptic connection between the SC and the injected region (RMTg in this study). The presence of either Cre-recombinase or mCherry within the borders of contralateral RMTg shows that indeed there is a monosynaptic connection between SC and contralateral RMTg (Fig. 4*B*,*C*). Moreover, the number of Cre-positive cells was greater in the contralateral than in the ipsilateral RMTg. The number of Cre-positive cells also increased caudally (Fig. 4*B*). The injection of the AAV2-EF1α-DIO-mCherry alone (without the AAV1-Syn-Cre-hGH injection into the SC) did not result in any expression of mCherry in the RMTg (data not shown). Moreover, the mCherry-positive axon terminals were present within the borders of both VTA and SNc (Fig. 4*D*). Axons were located mainly ipsilaterally to mCherry-positive RMTg, and their density was highest in the lateral VTA and medial SNc (Fig. 4*E*). These results indicate that RMTg neurons that are monosynaptically innervated by the contralateral SC are located preferentially in the caudal parts of RMTg and send ipsilateral projections directly to the midbrain dopaminergic system, with emphasis on the lateral VTA and medial SNc.

### SC stimulation activates predominantly RMTg neurons on the contralateral side

**Figure 5.**
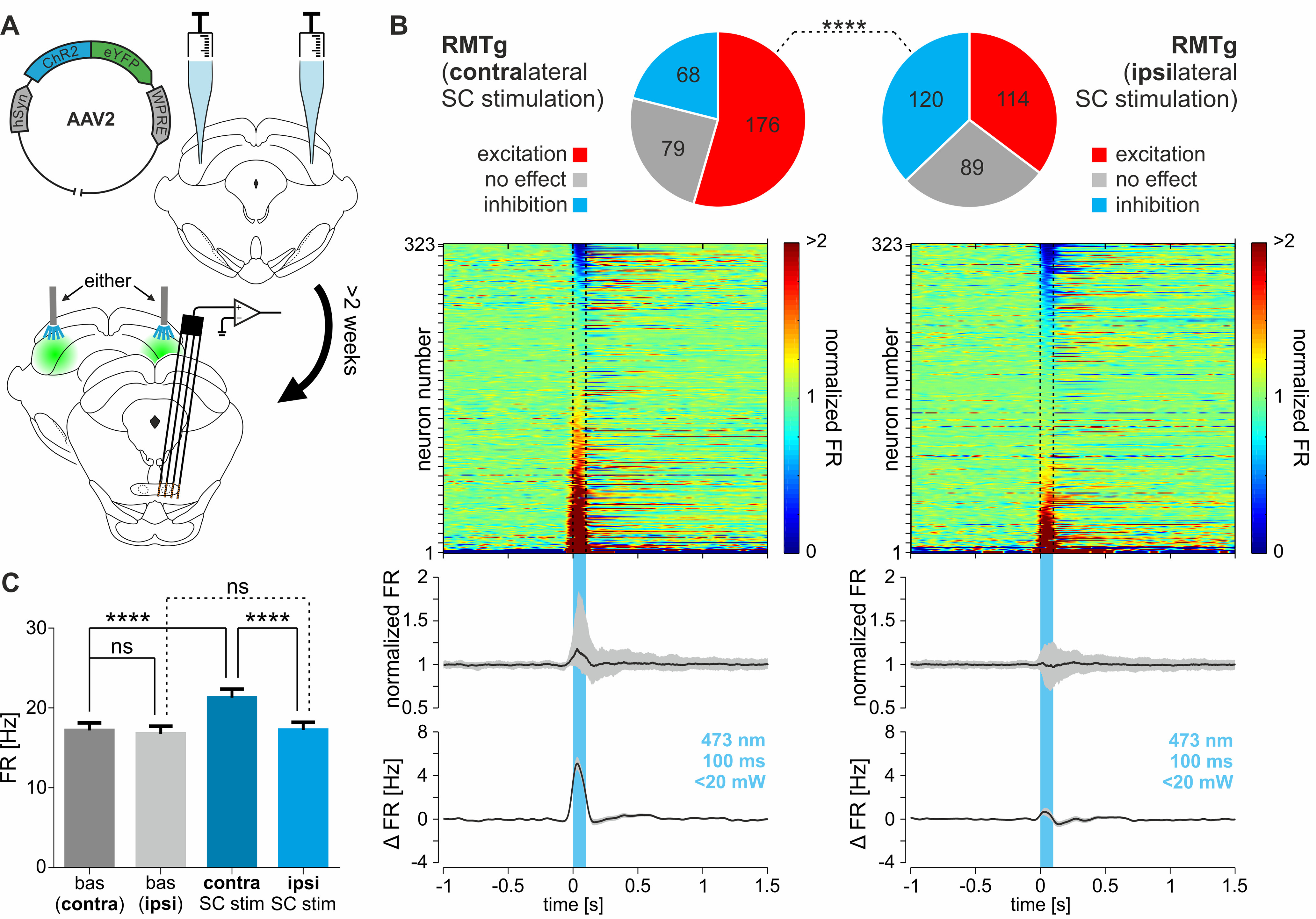
RMTg, at the population level, is excited by stimulation of the contralateral SC. ***A***, Scheme of the experiment. Bilateral SC injection of AAV-carrying genes for ChR2 and eYFP was performed. At least 2 weeks after the viral injections, the electrophysiological recordings of RMTg using a silicon probe were conducted while either contralateral or ipsilateral SC was optogenetically stimulated. ***B***, Top panels, Pie charts showing the proportions of responses (excitation, no effect, inhibition) of RMTg neurons elicited by optogenetic stimulation of either contralateral or ipsilateral SC. Middle panels, Peristimulus heatmaps showing the reactions of all recorded RMTg neurons (*n* = 323) to optogenetic stimulation of the contralateral or ipsilateral SC (responses, shown in rows, are sorted by the amplitude of the response during the stimulation). Bottom panels, Peristimulus median firing rate (normalized to baseline; interquartile range marked with gray color) and peristimulus mean change in firing rate (±SEM marked with gray color) of all recorded RMTg neurons during the stimulation of either contralateral or ipsilateral SC. Blue vertical bar indicates stimulation time (473 nm, 100 ms, <20 mW). ***C***, Firing rate (±SEM) of all recorded RMTg neurons during baseline and during optogenetic stimulation of contralateral or ipsilateral SC. ****p < 0.0001. ns, Nonsignificant.

The activity of 624 RMTg neurons was recorded in 19 rats from which 323 neurons underwent both contralateral and ipsilateral SC stimulation (Fig. 5*A*, scheme of experiments). Localization of each recorded neuron was confirmed through histologic verification. Neurons in the RMTg (*n* = 624) displayed a spontaneous firing rate of 20.14 ± 0.86 Hz. A similar firing rate was observed in the neurons that underwent both contralateral and ipsilateral SC stimulation (*n* = 323, 16.96 ± 0.97 Hz; *p* = 0.21, Mann–Whitney test). The spontaneous activity of RMTg neurons was similar to that described previously in the literature (Bourdy and Barrot, 2012).

Optogenetic stimulation of either contralateral or ipsilateral SC was repeated every 6 s (minimum, 14 stimulations; mean stimulation count ± SD, 92.1 ± 36.8; each cell was subjected to the same number of ipsilateral and contralateral SC stimulations). The vast majority of RMTg neurons were excited by the contralateral SC stimulation (176 of 323 neurons, 54.5%; Fig. 5*B*), after which some neurons were unresponsive (79 of 323, 24.5%; Fig. 5*B*) and only a small proportion of neurons was inhibited (68 of 323, 21%; Fig. 5*B*). In contrast, once the ipsilateral SC stimulation was performed, the number of inhibited neurons nearly doubled (120 of 323, 37.2%; Fig. 5*B*), the number of excited neurons clearly decreased (114 of 323, 35.3%; Fig. 5*B*), and the proportion of unresponsive neurons did not change (89 of 323, 27.5%; Fig. 5*B*). The distribution of RMTg neuron response types was clearly different between contralateral and ipsilateral SC stimulations (Fig. 5*B*; χ^2^_(2)_ = 28.23, *p* < 0.0001). At the population level, contralateral SC stimulation elevated the activity of RMTg neurons (median, 112% of baseline activity; first to third quartile, 94.11–173.7%; Fig. 5*B*; one-sample Wilcoxon signed-rank test, theoretical median: 100%; *n* = 323; *p* < 0.0001); however, the ipsilateral stimulation caused no effect (median, 100.2% of baseline activity; first to third quartile, 79.12–122.5%; Fig. 5*B*; one-sample Wilcoxon signed-rank test, theoretical median: 100%; *n* = 323; *p* = 0.53). The average increase of RMTg neuron firing caused by the stimulation of contralateral SC was 4.12 ± 0.53 Hz (one-sample Wilcoxon signed-rank test, theoretical median: 0 Hz; *n* = 323; *p* < 0.0001). In contrast, the stimulation of the ipsilateral SC did not change the activity of the RMTg neuron population (0.49 ± 0.33 Hz; one-sample Wilcoxon signed-rank test, theoretical median: 0 Hz; *n* = 323; *p* = 0.72). The overall firing rate of RMTg has changed only when the contralateral SC was stimulated (Fig. 5*C*; baseline before contralateral stimulation, 17.18 ± 0.97 Hz; baseline before ipsilateral stimulation, 16.74 ± 0.96 Hz; contralateral stimulation, 21.30 ± 1.06 Hz; ipsilateral stimulation, 17.23 ± 0.99 Hz; Friedman test, *n* = 323; Friedman statistic: 50.94, *p* < 0.0001; followed by Dunn's *post hoc* test: baseline before contralateral stimulation vs contralateral stimulation, *p* < 0.0001; contralateral stimulation vs ipsilateral stimulation, *p* < 0.0001). It is noteworthy that the inhibitory responses may be more difficult to detect because the dynamic range for excitation is larger than for inhibition. Nevertheless, the presented results indicate that the stimulation of the contralateral SC induces excitation at the level of the RMTg neuron population, as opposed to no effect during the stimulation of the ipsilateral SC.

**Figure 6.**
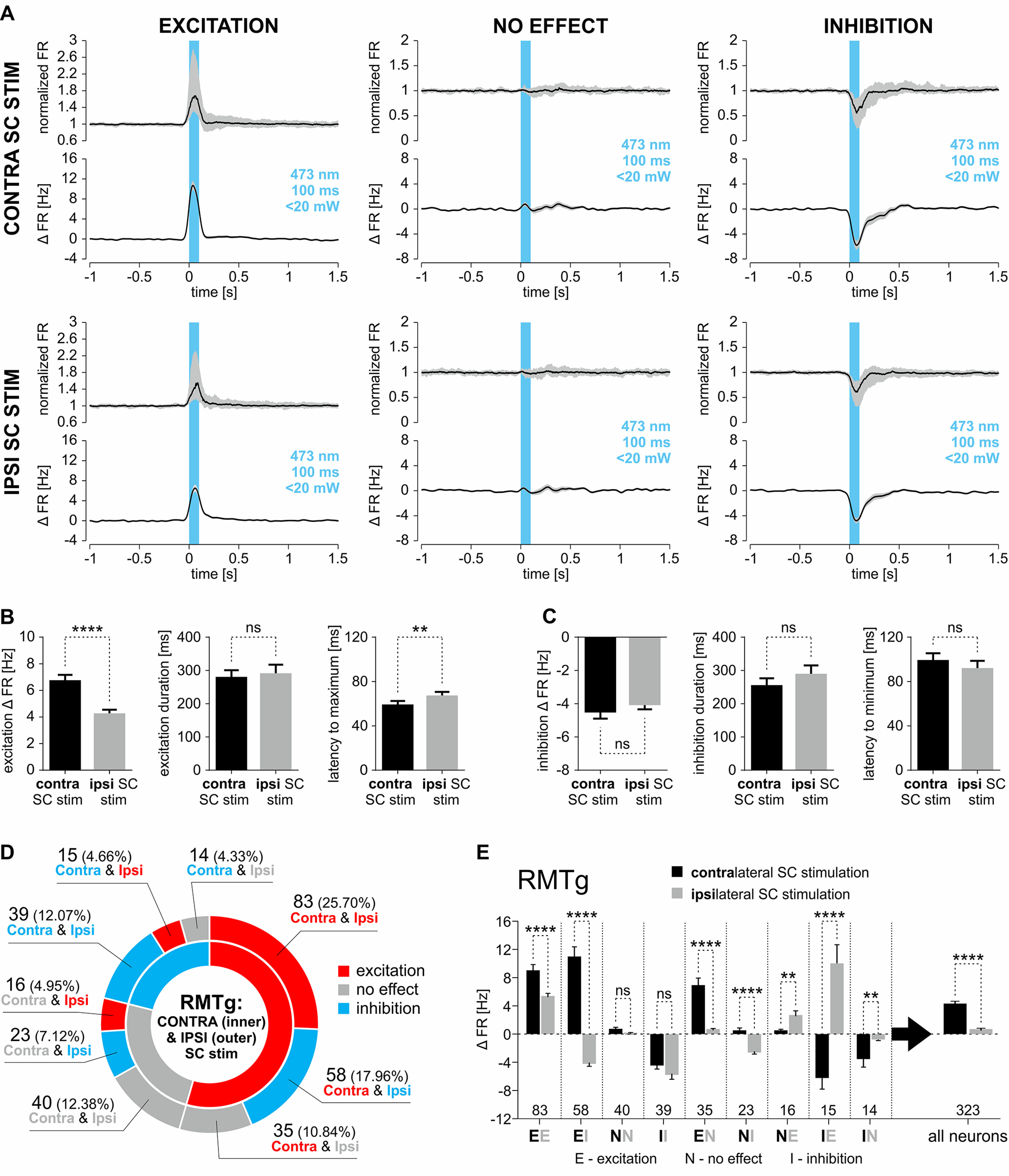
The comparison of the responses of RMTg neurons to optogenetic stimulation of either contralateral or ipsilateral SC. ***A***, Peristimulus median firing rate (normalized to baseline; interquartile range marked with gray color) and peristimulus mean change in firing rate (±SEM marked with gray color) of RMTg neurons that were either excited or inhibited or did not respond to the stimulation of contralateral (top) or ipsilateral (bottom) SC. The stimulation time is marked with a blue vertical bar (473 nm, 100 ms, <20 mW). ***B***, Parameters of responses of RMTg neurons that were excited by the stimulation of contralateral or ipsilateral SC. Left, Change in firing rate (±SEM). Middle, Duration of the excitatory response (±SEM). Right, Latency to maximum of the response. ***C***, Parameters of responses of all RMTg neurons that were inhibited by the stimulation of contralateral or ipsilateral SC. Left, Change in firing rate (±SEM). Middle, Duration of the inhibitory response (±SEM). Right, Latency to minimum of the response (±SEM). ***D***, Pie chart showing the proportions of RMTg neurons grouped based on combination of response types to the stimulation of both contralateral and ipsilateral SC. ***E***, Change in firing rate (±SEM) of RMTg neurons grouped based on their response (E, excitation; N, no effect; I, inhibition) to the stimulation of contralateral (black solid bars) and ipsilateral (gray solid bars) SC. **p < 0.01, ****p < 0.0001. ns, Nonsignificant.

Nevertheless, stimulation of either contralateral or ipsilateral SC can elicit mixed responses of RMTg neurons. The peristimulus median normalized firing rate and the average change in frequency of firing rate are depicted in Figure 6*A*, separately for RMTg neurons that were excited, unresponsive, or inhibited by stimulation of contralateral or ipsilateral SC. The parameters of response provided by automated statistical effect detection (for details, see Data analysis) were compared for both contralateral and ipsilateral SC stimulation. Stimulation of the contralateral SC evoked stronger excitation of RMTg neurons compared with the stimulation of ipsilateral SC (Fig. 6*B*; change in firing rate after contralateral SC stimulation, 6.69 ± 0.47 Hz; vs ipsilateral SC stimulation, 4.2 ± 0.34 Hz; Mann–Whitney test: number of RMTg cells that underwent contralateral SC stimulation (n_c_) = 176, ipsilateral SC stimulation (n_i_) = 114, U = 7198, *p* < 0.0001). There was no difference observed between inhibition induced by contralateral and ipsilateral SC stimulation (Fig. 6*C*; change in firing rate after contralateral SC stimulation, −4.47 ± 0.43 Hz; vs ipsilateral SC stimulation, −4.03 ± 0.31 Hz; Mann–Whitney test: n_c_ = 68, n_i_ = 120, U = 3757, *p* = 0.37). There was also no stimulation side-related difference in the duration of excitation (Fig. 6*B*; contralateral SC stimulation, 278 ± 29 ms; vs ipsilateral SC stimulation, 289 ± 33 ms; Mann–Whitney test: n_c_ = 176, n_i_ = 114, U = 9348, *p* = 0.33) or inhibition (Fig. 6*C*; contralateral SC stimulation, 253 ± 23 ms; vs ipsilateral SC stimulation, 287 ± 28 ms; Mann–Whitney test: n_c_ = 68, n_i_ = 120, U = 4043, *p* = 0.92) of RMTg neurons. The latency to peak effect was slightly shorter for contralateral than for ipsilateral SC stimulation in cases of excitation (Fig. 6*B*; contralateral SC stimulation, 58 ± 4 ms; vs ipsilateral SC stimulation, 66 ± 4 ms; Mann–Whitney test: n_c_ = 176, n_i_ = 114, U = 8208, *p* = 0.009), but not in cases of inhibition (Fig. 6*C*; contralateral SC stimulation, 98 ± 7 ms; vs ipsilateral SC stimulation, 91 ± 7 ms; Mann–Whitney test: n_c_ = 68, n_i_ = 120, U = 3471, *p* = 0.09).

Neurons were then divided into groups based on their reaction to both contralateral and ipsilateral SC stimulation. Most RMTg neurons were excited, no matter which side of the brain the SC stimulation was on (83 of 323 neurons, 25.7%; Fig. 6*D*). The second most numerous group of RMTg neurons comprised those that elevated their activity on contralateral SC stimulation and decreased their activity on ipsilateral SC stimulation (58 of 323 neurons, 18%; Fig. 6*D*). The second least numerous group of RMTg neurons comprised those inhibited by contralateral SC stimulation and excited by ipsilateral SC stimulation (15 of 323 neurons, 4.6%; Fig. 6*D*). The change in firing rate during the optogenetic stimulation period (first 100 ms from the stimulation onset) was assessed for all of the groups (divided according to reaction types to both contralateral and ipsilateral SC stimulation) and further analyzed (Fig. 6*E*). It turned out that the strongest excitation was observed in the group of RMTg neurons excited by contralateral and inhibited by ipsilateral SC stimulation.

### Spatial distribution of RMTg neurons excited, inhibited, or unresponsive to the optogenetic stimulation of the contralateral and ipsilateral SC

**Figure 7.**
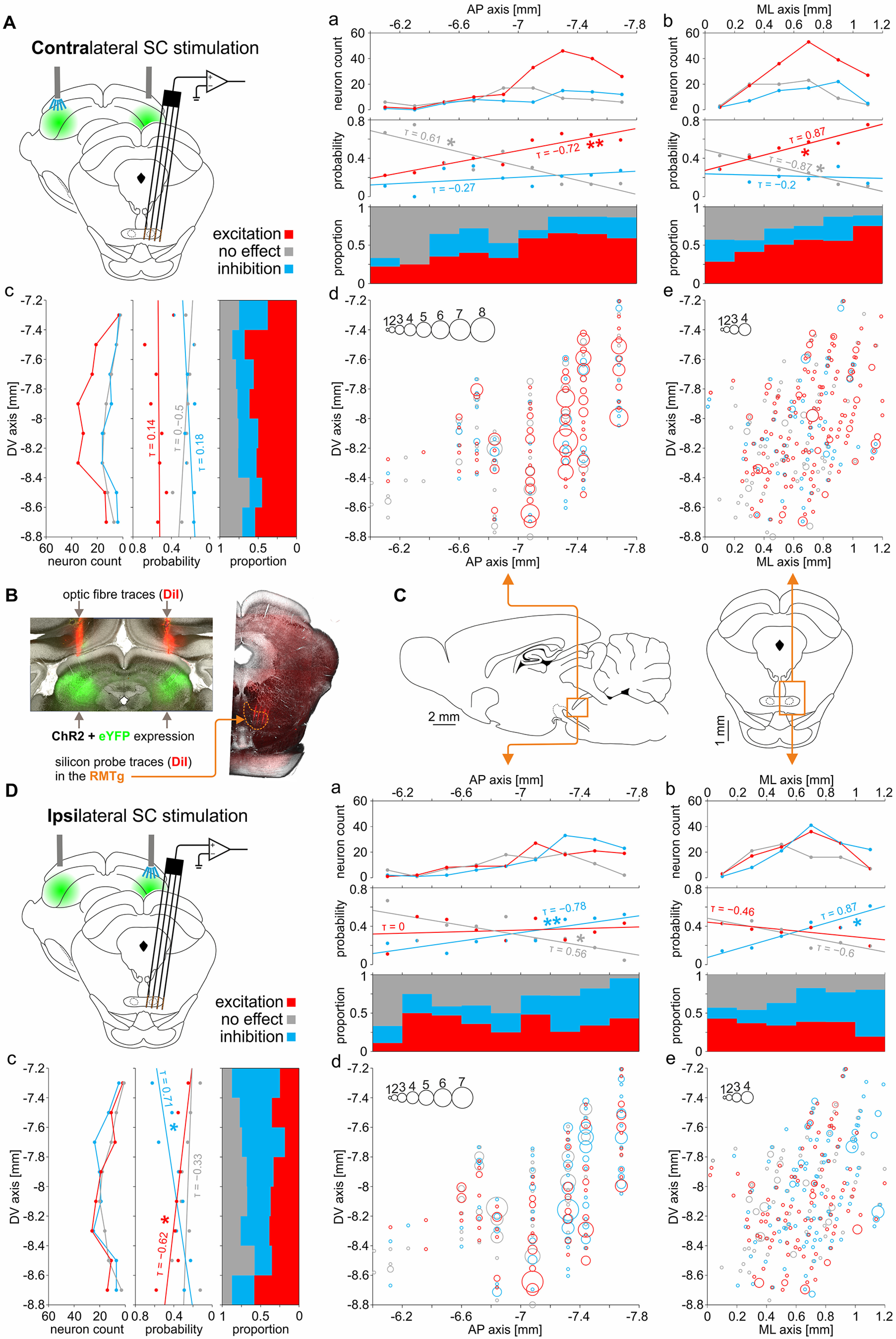
Spatial distribution of RMTg neurons excited, inhibited, or unresponsive to the optogenetic stimulation of the contralateral and ipsilateral SC. ***A***, ***d***, ***e***, Spatial distribution of the responses of RMTg neurons to stimulation of the contralateral SC, in the DV, the AP, and the ML axes. The number of neurons and the probability of encountering a neuron with a given response type, as well as the proportion of response types in the AP, ML, and DV axes, are shown in ***a–c***, respectively. ***B***, Exemplary images showing the position of the silicon probe shanks (DiI dye traces; red color) within the RMTg, and optical fibers placement (DiI dye traces; red color) above the ChR2-eYFP-expressing (green color) region of SC. The orange dashed line indicates the RMTg boundaries based on the anti-FoxP1 immunostaining. ***C***, Diagram of the brain, in both sagittal (left) and coronal (right) planes, with the area of RMTg used in the spatial distribution analysis, outlined. ***D***, ***d***, ***e***, Spatial distribution of the responses of RMTg neurons to stimulation of the ipsilateral SC, in the DV, the AP, and the ML axes. The number of neurons and the probability of encountering a neuron with a given response type, as well as the proportion response types in the AP, ML, and DV axes, are shown in ***a–c***, respectively. *p < 0.05, **p < 0.01.

The localization of all the recorded neurons, with color-coded type of response to SC stimulation, is shown in Figure 7. An example of DiI dye trace left by the MEA in the RMTg, as well as the eYFP expression in the SC and DiI dye traces left by the optical fibers implanted above the SC, is shown in Figure 7*B*. Spatial distribution of recorded RMTg neurons is depicted in either AP versus DV or ML versus DV axes (Fig. 7*C*). It was observed that spatial distribution of neurons that were excited, unresponsive, or inhibited by either contralateral or ipsilateral SC stimulation differs (contralateral SC stimulation: Pillai's trace = 0.11, *F*_(6,638)_ = 6.1, *p* < 0.0001, MANOVA; ipsilateral SC stimulation: Pillai's trace = 0.14, *F*_(6,638)_ = 7.7, *p* < 0.0001, MANOVA). Additionally, the distribution of observed response types (i.e., excitation, inhibition, no response) to SC stimulation significantly depended on the location of recorded RMTg neurons along the AP and ML axes (contralateral SC stimulation, AP axis: χ^2^_(16)_ = 47.03, *p* < 0.0001; ML axis: χ^2^_(10)_ = 25.67, *p* = 0.004; ipsilateral SC stimulation, AP axis: χ^2^_(16)_ = 48.71, *p* < 0.0001; ML axis: χ^2^_(10)_ = 30.29, df = 10, *p* = 0.0006; χ^2^ test), but not along the DV axis (contralateral SC stimulation, DV axis: χ^2^_(14)_ = 10.42, *p* = 0.7; ipsilateral SC stimulation, DV axis: χ^2^_(14)_ = 22.62, *p* = 0.07; χ^2^ test). Therefore, we conducted detailed correlation tests to determine the relationship between particular response types and spatial distribution in each axis. The probability of finding the RMTg neurons that are excited by the contralateral SC stimulation is highest in the caudal part of the structure (Fig. 7*A*,*a*,*d*; Kendall's tau-b correlation coefficient (τ_b_) = −0.72, p = 0.0059, Kendall's correlation). In contrast, in the caudal RMTg it was more likely to find neurons inhibited by the stimulation of the ipsilateral SC (Fig. 7*D*,*a*,*d*; τ_b_ = −0.78, *p* = 0.0028, Kendall's correlation). For both contralateral and ipsilateral SC stimulation, the probability of encountering an unresponsive RMTg neuron decreased as the recording position was more caudal [contralateral SC stimulation (Fig. 7*A*,*a*,*d*): τ_b_ = 0.61, *p* = 0.025, Kendall's correlation; ipsilateral SC stimulation (Fig. 7*D*,*a*,*d*): τ_b_ = 0.56, *p* = 0.047, Kendall's correlation]. Accordingly, the probability of finding a neuron that was inhibited by contralateral SC stimulation or excited by ipsilateral SC stimulation was uniformly distributed in the anteroposterior axis [contralateral SC stimulation (Fig. 7*A*,*a*,*d*) τ_b_ = −0.27, *p* = 0.36, Kendall's correlation; ipsilateral SC stimulation (Fig. 7*D*,*a*,*d*): τ_b_ = 0, *p* = 1, Kendall's correlation]. In the mediolateral axis, the probability of finding neurons excited by contralateral SC stimulation or inhibited by ipsilateral SC stimulation increased with increasing laterality [contralateral SC stimulation (Fig. 7*A*,*b*,*e*): τ_b_ = 0.86, *p* = 0.017, Kendall's correlation; ipsilateral SC stimulation (Fig. 7*D*,*b*,*e*): τ_b_ = 0.86, *p* = 0.017, Kendall's correlation]. This tendency can be attributed to the fact that the caudal parts of RMTg are also more lateral in the brain. Accordingly, the number of unresponsive neurons grew medially in the case of contralateral SC stimulation (Fig. 7*A*,*b*,*e*; τ_b_ = −0.87, *p* = 0.017, Kendall's correlation), but not in the case of ipsilateral SC stimulation (Fig. 7*D*,*b*,*e*; τ_b_ = −0.6, *p* = 0.14, Kendall's correlation). No mediolateral distribution trend was observed when it comes to neurons inhibited by the contralateral SC stimulation or excited by the ipsilateral SC stimulation [contralateral SC stimulation (Fig. 7*A*,*b*,*e*): τ_b_ = −0.2, *p* = 0.72, Kendall's correlation; ipsilateral SC stimulation (Fig. 7*D*,*b*,*e*): τ_b_ = −0.47, *p* = 0.27, Kendall's correlation]. On the other hand, RMTg neurons excited, inhibited, and nonresponsive to the contralateral SC stimulation were evenly distributed in the dorsoventral axis (Fig. 7*A*,*c–e*; excited: τ_b_ = 0.14, *p* = 0.72, Kendall's correlation; inhibited: τ_b_ = 0.18, *p* = 0.63, Kendall's correlation; nonresponsive: τ_b_ = −0.5, *p* = 0.11, Kendall's correlation). However, in the case of ipsilateral SC stimulation, more inhibited neurons were observed dorsally (Fig. 7*D*,*c–e*; τ_b_ = 0.71, *p* = 0.014, Kendall's correlation) and less excited neurons were observed ventrally (Fig. 7;*D*,*c–e* τ_b_ = −0.61, *p* = 0.042, Kendall's correlation), but no correlation was seen in the case of unresponsive cells (Fig. 7*D*,*c–e*; τ_b_ = −0.33, *p* = 0.33, Kendall's correlation). Again, this tendency can be explained by the fact that the position of the caudal parts of RMTg is more dorsal in the brain.

### Stimulation of RMTg neurons innervated by the contralateral SC inhibit VTA and SNc dopaminergic neurons

**Figure 8.**
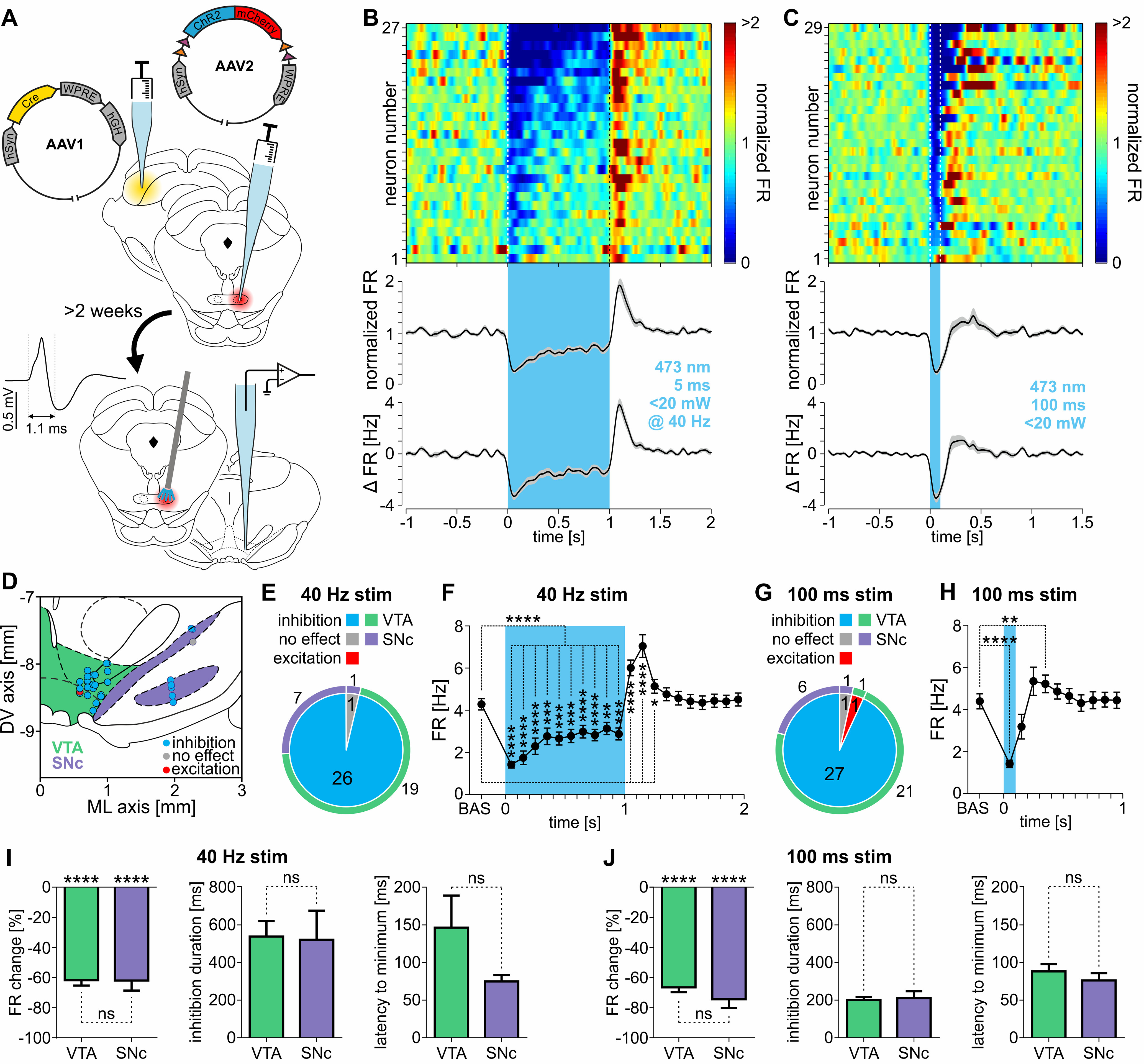
Stimulation of RMTg neurons innervated by the contralateral SC inhibit DA-like neurons in the VTA and SNc. ***A***, Scheme of the experiment. SC was unilaterally injected with the transsynaptic viral vector (AAV1-hSyn-Cre-hGH) carrying the gene for Cre recombinase and contralateral RMTg was injected with AAV2-EF1α-DIO-ChR2(H134R)-mCherry carrying Cre-dependent genes for ChR2 and mCherry fluorescent protein. At least 2 weeks later, extracellular recordings of the activity of midbrain DA-like neurons was conducted while RMTg neurons, monosynaptically innervated by the contralateral SC, were optogenetically stimulated. The shape of a typical action potential of a DA-like neuron is shown in the inset on the left side. ***B***, ***C***, Top panels, Heatmaps of peristimulus firing rates (normalized to baseline) showing the responses of all recorded DA-like neurons to RMTg stimulation with trains of laser light pulses (473 nm, <20 mW, 5 ms pulses at 40 Hz over 1 s; ***B***) or single laser light pulses (473 nm, 100 ms, <20 mW; ***C***). Responses of individual neurons are shown in rows and are sorted by the amplitude of the response during the stimulation time (marked by vertical dashed line). Bottom panels, Peristimulus mean firing rate (normalized to baseline; ±SEM marked with gray color) and peristimulus mean change in firing rate (±SEM marked with gray color). The stimulation time is marked with a blue vertical bar. ***D***, Localization of all recorded DA-like neurons with color-coded type of response to the optogenetic stimulation of RMTg neurons monosynaptically innervated by the contralateral SC. ***E***, ***G***, Pie charts showing the proportions of response types (inhibition, no effect, excitation) of VTA (marked by green outer ring) and SNc (marked by purple outer ring) DA-like neurons elicited by train (5 ms at 40 Hz; ***E***) or single-pulse (100 ms; ***G***) optogenetic stimulation of RMTg neurons innervated by the contralateral SC. ***F***, ***H***, Mean firing rates before, during, and after the train (5 ms at 40 Hz; ***F***) or single-pulse (100 ms; ***H***) optogenetic stimulation of RMTg neurons innervated by the contralateral SC (100 ms bins). ***I***, ***J***, From left to right: percentage change in firing rate, duration, and latency to the minimum of the response of DA-like neurons both in VTA (green bars) and SNc (purple bars) caused by train (5 ms at 40 Hz; ***I***) or single-pulse (100 ms; ***J***) optogenetic stimulation of RMTg neurons innervated by the contralateral SC. *p < 0.05, **p < 0.01, ***p < 0.001, ****p < 0.0001. ns, Nonsignificant.

To gain optogenetic control over the RMTg neurons that are monosynaptically innervated by the contralateral superior colliculus, rats underwent unilateral injections of transsynaptic viral vector AAV1-hSyn-Cre-hGH into the SC, and of AAV2-EF1α-DIO-ChR2(H134R)-mCherry into the contralateral RMTg. The dependence of the expression of channelrhodopsin-2 fused with mCherry on the presence of Cre recombinase in cell bodies was confirmed by the lack of a reporter protein in RMTg of animals that were injected only with the AAV2-EF1α-DIO-ChR2(H134R)-mCherry viral vector (no injection of AAV1-hSyn-Cre-hGH was made; data not shown). The activity of 31 DA-like neurons (from three rats) was extracellularly recorded within the borders of VTA and SNc, while ipsilateral RMTg cells, monosynaptically innervated by the contralateral SC, were optogenetically stimulated (Fig. 8*A*, scheme of the experiment, *D*, location of recorded neurons). Either 40 Hz stimulation (5 ms laser light pulses) over 1 s or a single 100 ms pulse stimulation was used. The 40 Hz stimulation was repeated every 10 s and a 100 ms pulse every 6 s (minimum, 19 stimulations per neuron; mean stimulation count ± SD, 68.2 ± 24.7). Stimulation of the RMTg neurons with 40 Hz protocol inhibited almost all DA-like neurons (26 of 27, 96.30%, Fig. 8*E*). The inhibition during the time of the optogenetic stimulation was strong (Fig. 8*B*,*F*; baseline, 4.29 ± 0.27 Hz; during stimulation, 2.54 ± 0.28 Hz; *t* = 7.26, df = 26, *p* < 0.0001, paired *t* test). In most cases, the RMTg stimulation-induced inhibition was followed by a significant rebound excitation (Fig. 8*B*,*F*; *F*_(3.02,78.4)_ = 43.06, *p* < 0.0001, repeated-measures ANOVA, followed by Dunnett's *post hoc* test). Similarly, 100 ms laser light pulses delivered to RMTg strongly inhibited most of the recorded DA-like neurons (27 of 29, 93.10%; Fig. 8*G*), while only one neuron was unresponsive and one was excited. Observed strong inhibition often outlasted the stimulation and was followed by rebound excitation (Fig. 8*C*,*H*; baseline, 4.39 ± 0.36 Hz; during stimulation, 1.42 ± 0.18 Hz; *F*_(3.41,95.55)_ = 17.79, *p* < 0.0001, repeated-measures ANOVA, followed by Dunnett's *post hoc* test). No difference in the strength of RMTg stimulation-induced inhibition was observed in the inhibited neurons between VTA and SNc DA-like neurons, both in the case of 5 ms at 40 Hz stimulation (Fig. 8*I*, left; VTA, −61.74 ± 3.56%; SNc, −62.02 ± 6.61%; *n*_VTA_ = 19, *n*_SNc_ = 7, *t* = 0.04, df = 24, *p* = 0.97, unpaired *t* test) and 100 ms single-pulse stimulation (Fig. 8*J*, left; VTA, −66.47 ± 3.23%; SNc, −74.42 ± 5.76%; *n*_VTA_ = 21, *n*_SNc_ = 6, *t* = 1.17, df = 25, *p* = 0.25, unpaired *t* test). Nevertheless, the change in activity in cases of both VTA and SNc was strong (Fig. 8*I*, left; VTA_40Hz_: −61.74 ± 3.56%, *t* = 17.37, df = 18, *p* < 0.0001; SNc_40Hz_: −62.02 ± 6.61%, *t* = 9.38, df = 6, *p* < 0.0001; VTA_100ms_: −66.47 ± 3.23%, *t* = 20.6, df = 20, *p* < 0.0001; SNc_100ms_: −74.42 ± 5.76%, *t* = 12.93, df = 5, *p* < 0.0001; one-sample *t* tests; theoretical mean, 0%). Additionally, no difference in the duration of inhibition of DA-like neurons in the VTA and SNc was observed, either in cases of 5 ms at 40 Hz stimulation (Fig. 8*I*, middle; VTA, 530.9 ± 82.8 ms; SNc, 520.4 ± 154.6 ms; U = 53, *p* = 0.44, Mann–Whitney test) or 100 ms single-pulse stimulation (Fig. 8*J*, middle; VTA, 200.6 ± 15.7 ms; SNc, 211.0 ± 36.6 ms; *t* = 0.30, df = 25, *p* = 0.77, unpaired *t* test). Similarly, the latency to minimum activity did not differ between VTA and SNc neurons both in cases of 5 ms at 40 Hz stimulation (Fig. 8*I*, right; VTA, 146.3 ± 42.7 ms; SNc, 74.9 ± 8.5 ms; U = 39, *p* = 0.12, Mann–Whitney test) and 100 ms single-pulse stimulation (Fig. 8*J*, right; VTA, 88.2 ± 9.6 ms; SNc, 76.2 ± 9.7 ms; U = 57, *p* = 0.74, Mann–Whitney test). Together, these results clearly show that RMTg neurons, which are innervated by the contralateral SC, can strongly inhibit the activity of DA-like neurons located in ipsilateral VTA and SNc.

### Stimulation of contralateral SC-originating axon terminals within the RMTg inhibits firing of the subset of DA-like VTA and SNc neurons

**Figure 9.**
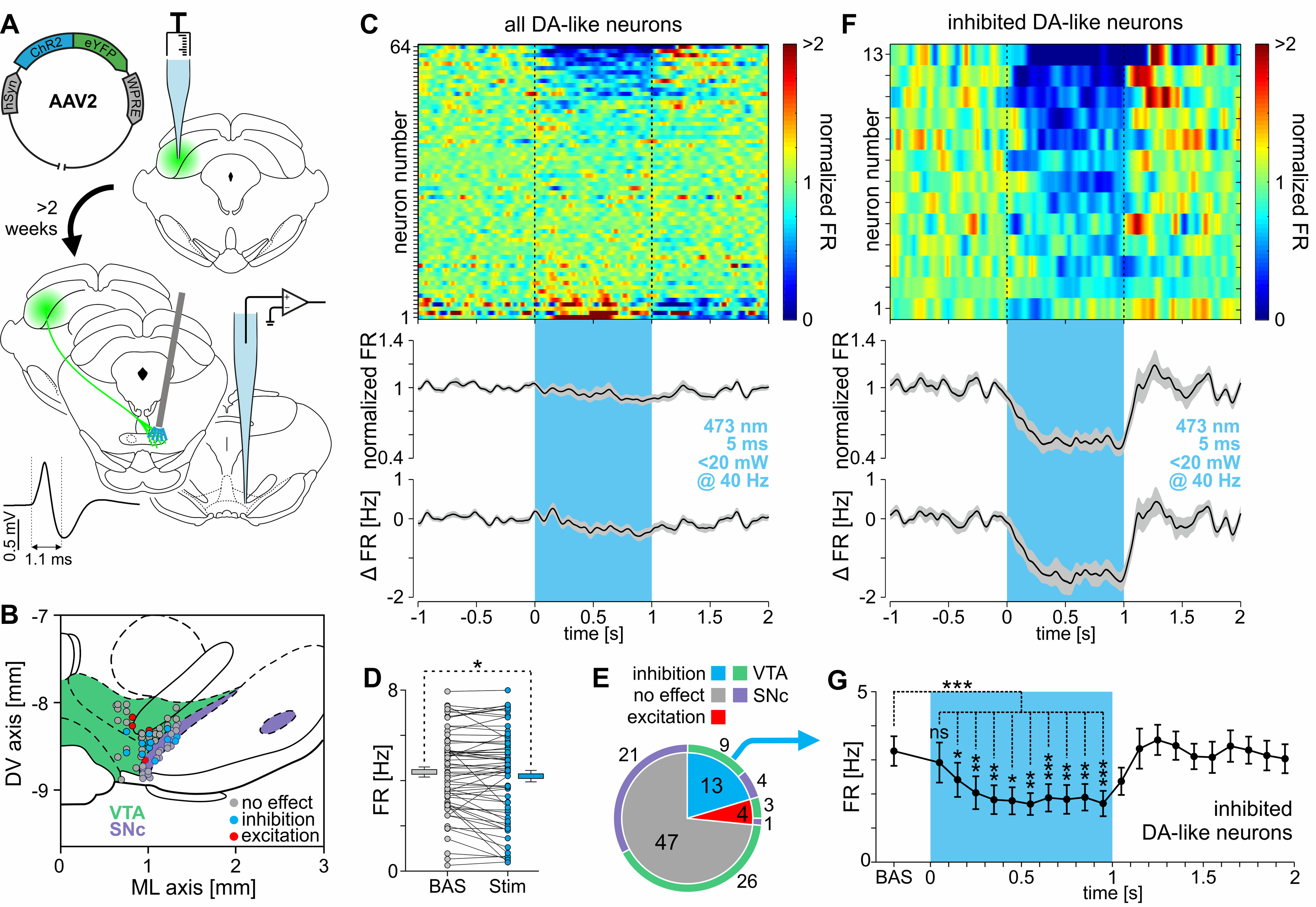
Stimulation of contralateral SC-originating axon terminals within the RMTg inhibits firing of the subset of DA-like VTA and SNc neurons. ***A***, Scheme of the experiment. SC was unilaterally injected with viral vector (AAV2-hSyn-ChR2-eYFP) carrying ChR2 and eYFP genes. At least 2 weeks later, extracellular recordings of the activity of the midbrain DA-like neurons were conducted while axon terminals descending from the contralateral SC were optogenetically stimulated in the RMTg. The shape of a typical action potential of a DA-like neuron is shown on the inset on the left side. ***B***, Localization of all recorded DA-like neurons with color-coded type of response to the optogenetic stimulation of SC-originating axon terminals within the RMTg. ***C***, Top, Heatmap of peristimulus normalized firing rates showing the response of all recorded DA-like neurons to the stimulation (5 ms at 40 Hz over 1 s) of the contralateral SC-originating axon terminals in the RMTg. Responses of individual neurons are shown in rows and are sorted by the amplitude of the response during the stimulation time (marked by vertical dashed lines). Bottom, Peristimulus mean normalized firing rate (±SEM marked with gray color) and peristimulus mean change in firing rate (±SEM marked with gray color). The stimulation time is marked with a blue vertical bar (473 nm, <20 mW, 5 ms pulses at 40 Hz over 1 s). ***D***, Mean firing rate of all recorded DA-like neurons before and during the stimulation. ***E***, Pie chart showing the proportion of responses (no effect, inhibition, excitation) of VTA (marked by green outer ring) and SNc (marked by purple outer ring) DA-like neurons elicited by the optogenetic stimulation of the contralateral SC-originating axons within the RMTg. ***F***, Top, Heatmap of peristimulus normalized firing rates showing the response of DA-like neurons that were inhibited by the stimulation of contralateral SC-originating axon terminals in the RMTg. Responses of individual neurons are shown in rows and are sorted by the amplitude of the response during the stimulation time (marked by vertical dashed lines). Bottom, Peristimulus mean normalized firing rate (±SEM marked with gray color) and peristimulus mean change in firing rate (±SEM marked with gray color). The stimulation time is marked with a blue vertical bar (473 nm, <20 mW, 5 ms pulses at 40 Hz over 1 s). ***G***, Mean firing rate of inhibited only DA-like neurons before, during, and after the optogenetic stimulation of the contralateral SC-originating axons within the RMTg (100 ms bins). *p < 0.05, **p < 0.01, ***p < 0.001. ns, Nonsignificant.

This subset of experiments used rats that underwent unilateral injection of AAV2-hSyn-ChR2(H134R)-eYFP into the SC. The activity of 64 DA-like neurons was recorded in the VTA and SNc from four rats (Fig. 9*B*, localization of the recorded neurons). During the recordings, channelrhodopsin-2-expressing axon terminals descending from the contralateral SC were stimulated within the borders of the RMTg ipsilaterally to the recorded DA-like neurons (Fig. 9*A*, scheme of the experiment) using 5 ms laser light pulses applied at 40 Hz for 1 s, repeated at least 15 times every 10 s (mean stimulation train count per recorded neuron ± SD, 89.4 ± 31.1). At the level of the whole population of recorded DA-like neurons, a small yet significant decrease of the electrical activity was observed during the stimulation (Fig. 9*C*,*D*; baseline, 4.38 ± 0.22 Hz; during stimulation, 4.2 ± 0.25 Hz; *t* = 2.05, df = 63, *p* = 0.045, paired *t* test). The stimulation of SC-originating axon terminals within the RMTg-induced inhibitory responses in 20.31% of all the recorded DA-like neurons (13 of 64; Fig. 9*E*) and excited only a small proportion of recorded neurons (6.25%, 4 of 64; Fig. 9*E*). The majority of the cells, however, were unresponsive to the stimulation (73.44%, 47 of 64; Fig. 9*E*). The proportion of response types was similar across VTA and SNc (Fig. 9*E*; χ^2^_(2)_ = 1.25, *p* = 0.54). Analysis of only those DA-like neurons whose activity was inhibited by the stimulation (Fig. 9*F*,*G*; baseline, 3.26 ± 0.43 Hz; during stimulation, 2.01 ± 0.40 Hz; *p* = 0.0002, Wilcoxon matched-pairs signed-rank test) showed that their response was delayed and increased gradually during the first 300 ms of stimulation, and no rebound excitation was observed after its termination (Fig. 9*G*; *F*_(4.05,48.57)_ = 9.76, *p* < 0.0001, repeated-measures ANOVA, followed by Dunnett's *post hoc* test). These results clearly show that excitation of the contralateral SC-originating axon terminals within the RMTg can activate, plausibly GABAergic, output of this structure, which in turn inhibits a subset of the midbrain DA-like neurons.

## Discussion

The presented results show anatomically and electrophysiologically that RMTg is a relay between the SC and midbrain dopaminergic system. Using different tract-tracing methods, we have revealed that RMTg neurons are monosynaptically innervated predominantly by the lateral intermediate layer of the contralateral SC. This anatomic asymmetry of innervation was reflected in the observed increase in RMTg neuronal population activity in response to optogenetic stimulation of contralateral but not ipsilateral SC. Furthermore, we have shown that activating elements of the neuronal pathway connecting SC with contralateral RMTg has an inhibitory impact on midbrain dopaminergic neurons. Together, it appears that SC can inhibit the contralateral dopaminergic system using an indirect pathway via contralateral RMTg.

Dopaminergic system integrates a wide range of information, including that from various sensory modalities. Resulting activity of dopaminergic neurons influences the behavior of animals, in particular the orienting and approach toward external stimuli. SC is one of the dominant sources of low-processed sensorial information (predominantly visual) reaching the midbrain dopaminergic system, as described in the Introduction. Previous studies have focused mainly on the innervation from the ipsilateral SC to dopaminergic neurons, sometimes using the presentation of stimuli in the receptive field contralateral to the examined DA neurons (which activates contralateral SC). However, with the discovery that RMTg is a source of strong inhibitory input to dopaminergic neurons (Jhou et al., 2009a; Bourdy and Barrot, 2012), the reports that this tegmental area might be innervated by contralateral SC (Kaufling et al., 2009; Jhou et al., 2009b; Yetnikoff et al., 2015) gained importance, allowing us to hypothesize that SC might drive DA neurons on both sides of the brain in the opposite direction.

Indeed, our tract-tracing experiments confirmed previous anatomic observations about neuronal projection from SC to contralateral RMTg and further described this connection in detail. Anterograde tracing revealed that axon terminals descending from SC are present within the borders of contralateral, but not ipsilateral, RMTg. These results were further corroborated by the retrograde tracing experiments, which additionally revealed that this neuronal pathway predominantly originates from the deeper SC layers, with emphasis on lateral parts of the intermediate layer of the contralateral SC. Moreover, transsynaptic tracing experiments showed that this connection is monosynaptic, excluding the possibility that SC-originating axons present in the contralateral RMTg (anterograde experiments) are just passing axons of the predorsal bundle. Nevertheless, the course and origin of the described neuronal pathway connecting SC with contralateral RMTg is similar to that of the predorsal bundle, which raises the possibility that observed neuronal fibers are collaterals of the tectospinal tract (Redgrave et al., 1986). Additionally, transsynaptic tracing experiments revealed that the subset of RMTg neurons innervated by the contralateral RMTg innervate midbrain dopaminergic system, with the emphasis on medial SNc and lateral VTA. It is hypothesized that this part of the dopaminergic system preferentially contains neurons that code motivational value (Bromberg-Martin et al., 2010). Nevertheless, a high density of RMTg axons was also present in other parts of the dopaminergic system, especially the lateral SNc, which is hypothesized to contain mainly neurons coding motivational salience. However, this intriguing issue is beyond the scope of this study. Nonetheless, we also found that SC preferentially innervates caudal RMTg, which is thought to innervate SNc (Smith et al., 2019). Together, this suggests that the output of RMTg neurons innervated by the contralateral SC may be slightly biased toward SNc.

Our results have also shown that unilateral activation of SC excites contralateral RMTg neurons at the level of neuronal population, leaving the activity of ipsilateral RMTg unchanged. Nevertheless, at the level of single neurons, mixed responses could be observed. Although, in cases of excited neurons, contralateral stimulation caused a stronger and faster response than ipsilateral stimulation. Finally, we have shown that both activating RMTg neurons that are monosynaptically innervated by the contralateral SC and activating contralateral SC-originating axons within the RMTg inhibit DA-like neurons in the VTA and SNc. It is noteworthy that direct activation of RMTg neurons innervated by the contralateral SC evoked stronger and more consistent reaction of midbrain DA-like neurons than did the stimulation of contralateral SC-originating axons in the RMTg. The difference may result from the fact that in the latter case the signal must travel through an extra synapse (SC→RMTg), thus relying its transmission on the responsiveness of the postsynaptic cell, which may be additionally lowered by anesthesia (Daló and Hackman, 2013).

As previous studies have described, the SC can increase the activity of dopaminergic neurons on the ipsilateral side of the brain. Our results showed that SC can also inhibit dopaminergic neurons on the contralateral side of the brain, via RMTg. Since SC receives visual information from the contralateral visual hemifield, a potentially salient and/or rewarding stimulus appearing on one body side may increase the activity of the contralateral dopaminergic neurons (directly), at the same time inhibiting the activity of dopaminergic neurons located ipsilaterally to the stimulus (via RMTg). Such a neuronal mechanism may underlie the recently described phenomenon, namely that dopaminergic neurons are not only excited by the contralateral rather than the ipsilateral sensory cues, but that there is also a small, yet visible, tendency of DA neurons to decrease their activity when an ipsilateral cue is presented (Engelhard et al., 2019). The proposed brain wiring may have behavioral consequences, since the direction of the movement animals is a manifestation of the difference in the activity of the left and right dopaminergic systems (Molochnikov and Cohen, 2014). The preferred side to which rats rotate or choose to move is contralateral to the hemisphere with higher striatal dopamine concentration (Zimmerberg et al., 1974; Glick et al., 1988). Also, dopamine injected unilaterally into the dorsal striatum biases the movements of rats toward the contralateral side (Joyce et al., 1981), and unilateral lesioning of dopaminergic neurons induces ipsiversive rotations (Arbuthnott and Crow, 1971; Iwamoto et al., 1976). Such rotations become even more frequent after amphetamine-induced dopamine elevation, since it happens only in the intact hemisphere. Accordingly, unilateral disinhibition of the dopaminergic system by RMTg lesion leads to contraversive body rotations (Bourdy et al., 2014; Barrot et al., 2016; Faivre et al., 2020). The basis for these phenomena is high lateralization of the dopaminergic system (i.e., the vast majority of dopaminergic fibers innervate ipsilaterally located striatum; Molochnikov and Cohen, 2014).

Each SC plays a key role in controlling eye, head, limb, and body movements in a direction contralateral to its location (Gandhi and Katnani, 2011; Villalobos and Basso, 2020). The intermediate SC layer, where we have found the most neurons innervating contralateral RMTg, plays a key role in motor guidance. Moreover, this layer has been shown to contain the most neurons innervating ipsilateral SNc and VTA (Comoli et al., 2003; Coizet et al., 2007; May et al., 2009; Yetnikoff et al., 2015). The fact that we have observed most RMTg-innervating SC neurons in the lateral SC is consistent with the fact that lateral SC receives information from the lower visual field and promotes contralateral approach behavior (e.g., foraging), as opposed to medial SC parts, which receive information from the upper visual field and promote avoidance or freezing (defense-like response; Sahibzada et al., 1986; Comoli et al., 2012). Accordingly, VTA DA neurons encourage approach toward important stimuli and rewards, and SNc DA neurons promote increased vigor and movement (Adamantidis et al., 2011; Saunders et al., 2018). Moreover, it was shown that a predator-like upper visual stimulus causes the activation of these SC neurons that preferentially innervate VTA GABA and not DA neurons, and that such connection promotes escape and not approach behavior (Zhou et al., 2019). Recently, another intriguing observation has been made: that preference of the reaction of DA neurons to contralateral sensory cues develops in the later training phases (Engelhard et al., 2019). It can be assumed that at the beginning of training, the animal actively scans the environment for possible patterns, receiving numerous stimuli of similar value, which would be reflected by disordered SC responses and thus similarly disordered responses of DA neurons. However, once the animal is trained, it orients toward the particular significant environmental cue, which could translate into a more stable pattern of activity in the circuits, both directly and indirectly via RMTg, connecting the SC with the dopaminergic system. This, in turn, can make the behavioral reaction of the animal quick and precisely directed.

In conclusion, the elevated SC activity may cause opposing effects in dopaminergic neurons located on both sides of the brain. Unilaterally activated SC (e.g., by lateralized sensory stimulus) can increase the firing of DA neurons located ipsilaterally, thereby promoting contralateral movement. Simultaneously, the same SC can inhibit the firing of contralateral DA neurons via increased activity of contralateral RMTg, therefore promoting ipsilateral, in relation to inhibited DA neurons, movement (Fig. 1). Since SC receives sensory information from the contralateral body side, both mechanisms would promote movement toward the side where sensory information appeared. Importantly, both processes, by increasing the disproportion of striatal dopamine release between hemispheres, are functionally synergistic and may efficiently elicit directed movement. Thus, the discussed brain wiring can serve as a potential mechanism contributing to behavioral lateralization dependent on the direction of incoming sensory information.

## References

[B1] Adamantidis AR, Tsai HC, Boutrel B, Zhang F, Stuber GD, Budygin EA, Touriño C, Bonci A, Deisseroth K, de Lecea L (2011) Optogenetic interrogation of dopaminergic modulation of the multiple phases of reward-seeking behavior. J Neurosci 31:10829–10835. 10.1523/JNEUROSCI.2246-11.2011 21795535 PMC3171183

[B2] Arbuthnott GW, Crow TJ (1971) Relation of contraversive turning to unilateral release of dopamine from the nigrostriatal pathway in rats. Exp Neurol 30:484–491. 10.1016/0014-4886(71)90149-x 5554236

[B3] Baik JH (2013) Dopamine signaling in reward-related behaviors. Front Neural Circuits 7:152. 10.3389/fncir.2013.00152 24130517 PMC3795306

[B301] Barrot M, Georges F, Veinante P (2016) The Tail of the Ventral Tegmental Area/Rostromedial Tegmental Nucleus: A Modulator of Midbrain Dopamine Systems. In: Handbook of Behavioral Neuroscience, pp 495–511. Elsevier B.V.

[B4] Bertram C, Dahan L, Boorman LW, Harris S, Vautrelle N, Leriche M, Redgrave P, Overton PG (2014) Cortical regulation of dopaminergic neurons: role of the midbrain superior colliculus. J Neurophysiol 111:755–767. 10.1152/jn.00329.2013 24225541 PMC3921396

[B5] Bourdy R, Barrot M (2012) A new control center for dopaminergic systems: pulling the VTA by the tail. Trends Neurosci 35:681–690. 10.1016/j.tins.2012.06.007 22824232

[B7] Bourdy R, Sánchez-Catalán MJ, Kaufling J, Balcita-Pedicino JJ, Freund-Mercier MJ, Veinante P, Sesack SR, Georges F, Barrot M (2014) Control of the nigrostriatal dopamine neuron activity and motor function by the tail of the ventral tegmental area. Neuropsychopharmacology 39:2788–2798. 10.1038/npp.2014.129 24896615 PMC4200489

[B6] Bromberg-Martin ES, Matsumoto M, Hikosaka O (2010) Dopamine in motivational control: rewarding, aversive, and alerting. Neuron 38:815–834. 10.1016/j.neuron.2010.11.022 PMC3032992 21144997

[B8] Coizet V, Comoli E, Westby GWM, Redgrave P (2003) Phasic activation of substantia nigra and the ventral tegmental area by chemical stimulation of the superior colliculus: an electrophysiological investigation in the rat. Eur J Neurosci 17:28–40. 10.1046/j.1460-9568.2003.02415.x 12534966

[B9] Coizet V, Overton PG, Redgrave P (2007) Collateralization of the tectonigral projection with other major output pathways of superior colliculus in the rat. J Comp Neurol 500:1034–1049. 10.1002/cne.21202 17183537 PMC3124759

[B10] Comoli E, Coizet V, Boyes J, Bolam JP, Canteras NS, Quirk RH, Overton PG, Redgrave P (2003) A direct projection from superior colliculus to substantia nigra for detecting salient visual events. Nat Neurosci 6:974–980. 10.1038/nn1113 12925855

[B11] Comoli E, Favaro PDN, Vautrelle N, Leriche M, Overton PG, Redgrave P (2012) Segregated anatomical input to sub-regions of the rodent superior colliculus associated with approach and defense. Front Neuroanat 6:9. 22514521 10.3389/fnana.2012.00009 PMC3324116

[B12] Daló NL, Hackman JC (2013) The anesthetic urethane blocks excitatory amino acid responses but not GABA responses in isolated frog spinal cords. J Anesth 27:98–103. 10.1007/s00540-012-1466-7 22926419

[B13] Dommett E, Coizet V, Blaha CD, Martindale J, Lefebvre V, Walton N, Mayhew JEW, Overton PG, Redgrave P (2005) How visual stimuli activate dopaminergic neurons at short latency. Science 307:1476–1479. 10.1126/science.1107026 15746431

[B14] Engelhard B, Finkelstein J, Cox J, Fleming W, Jang HJ, Ornelas S, Koay SA, Thiberge SY, Daw ND, Tank DW, Witten IB (2019) Specialized coding of sensory, motor and cognitive variables in VTA dopamine neurons. Nature 570:509–513. 10.1038/s41586-019-1261-9 31142844 PMC7147811

[B311] Faivre F, Sánchez-Catalán MJ, Dovero S, Bido S, Joshi A, Bezard E, Barrot M (2020) Ablation of the tail of the ventral tegmental area compensates symptoms in an experimental model of Parkinson's disease. Neurobiol Dis 139:104818. 32087289 10.1016/j.nbd.2020.104818

[B15] Gandhi NJ, Katnani HA (2011) Motor functions of the superior colliculus. Annu Rev Neurosci 34:205–231. 10.1146/annurev-neuro-061010-113728 21456962 PMC3641825

[B16] Glick SD, Carlson JN, Baird JL, Maisonneuve IM, Bullock AE (1988) Basal and amphetamine-induced asymmetries in striatal dopamine release and metabolism: bilateral in vivo microdialysis in normal rats. Brain Res 473:161–164. 10.1016/0006-8993(88)90329-0 3208119

[B17] Grace AA, Bunney BS (1980) Nigral dopamine neurons: intracellular recording and identification with L-dopa injection and histofluorescence. Science 210:654–656. 10.1126/science.7433992 7433992

[B18] Grace AA, Bunney BS (1983) Intracellular and extracellular electrophysiology of nigral dopaminergic neurons—1. Identification and characterization. Neuroscience 10:301–315, 307–315. 10.1016/0306-4522(83)90135-5 6633863

[B19] Huang L, Xi Y, Peng Y, Yang Y, Huang X, Fu Y, Tao Q, Xiao J, Yuan T, An K, Zhao H, Pu M, Xu F, Xue T, Luo M, So KF, Ren C (2019) A visual circuit related to habenula underlies the antidepressive effects of light therapy. Neuron 102:128–142.e8. 10.1016/j.neuron.2019.01.037 30795900

[B20] Iwamoto ET, Loh HH, Way EL (1976) Circling behavior in rats with 6-hydroxydopamine or electrolytic nigral lesions. Eur J Pharmacol 37:339–356. 10.1016/0014-2999(76)90042-x 986305

[B21] Jhou TC, Fields HL, Baxter MG, Saper CB, Holland PC (2009a) The rostromedial tegmental nucleus (RMTg), a GABAergic afferent to midbrain dopamine neurons, encodes aversive stimuli and inhibits motor responses. Neuron 61:786–800. 10.1016/j.neuron.2009.02.001 19285474 PMC2841475

[B22] Jhou TC, Geisler S, Marinelli M, Degarmo BA, Zahm DS (2009b) The mesopontine rostromedial tegmental nucleus: a structure targeted by the lateral habenula that projects to the ventral tegmental area of Tsai and substantia nigra compacta. J Comp Neurol 513:566–596. 10.1002/cne.21891 19235216 PMC3116663

[B23] Joyce JN, Davis RE, Van Hartesveldt C (1981) Behavioral effects of unilateral dopamine injection into dorsal or ventral striatum. Eur J Pharmacol 72:1–10. 10.1016/0014-2999(81)90290-9 7196334

[B24] Kaufling J, Veinante P, Pawlowski SA, Freund-Mercier MJ, Barrot M (2009) Afferents to the GABAergic tail of the ventral tegmental area in the rat. J Comp Neurol 513:597–621. 10.1002/cne.21983 19235223

[B25] May PJ (2006) The mammalian superior colliculus: laminar structure and connections. Prog Brain Res 151:321–378. 10.1016/S0079-6123(05)51011-2 16221594

[B26] May PJ, McHaffie JG, Stanford TR, Jiang H, Costello MG, Coizet V, Hayes LM, Haber SN, Redgrave P (2009) Tectonigral projections in the primate: a pathway for pre-attentive sensory input to midbrain dopaminergic neurons. Eur J Neurosci 29:575–587. 10.1111/j.1460-9568.2008.06596.x 19175405 PMC2856337

[B27] Mogenson GJ, Jones DL, Yim CY (1980) From motivation to action: functional interface between the limbic system and the motor system. Prog Neurobiol 14:69–97. 10.1016/0301-0082(80)90018-0 6999537

[B28] Molochnikov I, Cohen D (2014) Hemispheric differences in the mesostriatal dopaminergic system. Front Syst Neurosci 8:110. 10.3389/fnsys.2014.00110 24966817 PMC4052732

[B29] Pachitariu M, Steinmetz N, Kadir S, Carandini M, Harris KD (2016) Kilosort: realtime spike-sorting for extracellular electrophysiology with hundreds of channels. doi: 10.1101/061481.

[B30] Paxinos G, Watson C (2007) The rat brain in stereotaxic coordinates, Ed 6. Amsterdam: Elsevier. 10.1016/0165-0270(80)90021-7 6110810

[B31] Prévost-Solié C, Contestaile A, Musardo S, Huber C, Bariselli S, Carleton A, Bellone C (2019) Superior colliculus to VTA pathway controls orienting behavior during conspecific interaction. bioRxiv. doi: 10.1101/735340. PMC8831635 35145124

[B32] Redgrave P, Gurney K (2006) The short-latency dopamine signal: a role in discovering novel actions? Nat Rev Neurosci 7:967–975. 10.1038/nrn2022 17115078

[B33] Redgrave P, Odekunle A, Dean P (1986) Tectal cells of origin of predorsal bundle in rat: location and segregation from ipsilateral descending pathway. Exp Brain Res 63:279–293. 10.1007/BF00236845 3093259

[B34] Redgrave P, Coizet V, Comoli E, McHaffie JG, Leriche M, Vautrelle N, Hayes LM, Overton P (2010) Interactions between the midbrain superior colliculus and the basal ganglia. Front Neuroanat 4:132. 20941324 10.3389/fnana.2010.00132 PMC2952460

[B35] Sahibzada N, Dean P, Redgrave P (1986) Movements resembling orientation or avoidance elicited by electrical stimulation of the superior colliculus in rats. J Neurosci 6:723–733. 10.1523/JNEUROSCI.06-03-00723.1986 3958791 PMC6568468

[B36] Saunders BT, Richard JM, Margolis EB, Janak PH (2018) Dopamine neurons create Pavlovian conditioned stimuli with circuit-defined motivational properties. Nat Neurosci 21:1072–1083. 10.1038/s41593-018-0191-4 30038277 PMC6082399

[B37] Smith RJ, Vento PJ, Chao YS, Good C, Jhou TC (2019) Gene expression and neurochemical characterization of the rostromedial tegmental nucleus (RMTg) in rats and mice. Brain Struct Funct 224:219–238. 10.1007/s00429-018-1761-7 30302539 PMC6467516

[B38] Takakuwa N, Kato R, Redgrave P, Isa T (2017) Emergence of visually-evoked reward expectation signals in dopamine neurons via the superior colliculus in V1 lesioned monkeys. Elife 6:e24459. 10.7554/eLife.24459 28628005 PMC5529105

[B39] Ungless MA, Grace AA (2012) Are you or aren't you? Challenges associated with physiologically identifying dopamine neurons. Trends Neurosci 35:422–430. 10.1016/j.tins.2012.02.003 22459161 PMC3383926

[B40] Villalobos CA, Basso MA (2020) Optogenetic activation of the inhibitory nigro-collicular circuit evokes orienting movements in mice. bioRxiv. doi: 10.1101/2020.05.21.107680. PMC10144672 35443172

[B41] Watabe-Uchida M, Zhu L, Ogawa SK, Vamanrao A, Uchida N (2012) Whole-brain mapping of direct inputs to midbrain dopamine neurons. Neuron 74:858–873. 10.1016/j.neuron.2012.03.017 22681690

[B42] Wise RA (2004) Dopamine, learning and motivation. Nat Rev Neurosci 5:483–494. 10.1038/nrn1406 15152198

[B43] Yetnikoff L, Cheng AY, Lavezzi HN, Parsley KP, Zahm DS (2015) Sources of input to the rostromedial tegmental nucleus, ventral tegmental area, and lateral habenula compared: a study in rat. J Comp Neurol 523:2426–2456. 10.1002/cne.23797 25940654 PMC4575621

[B44] Zhou Z, Liu X, Chen S, Zhang Z, Liu Y, Montardy Q, Tang Y, Wei P, Liu N, Li L, Song R, Lai J, He X, Chen C, Bi G, Feng G, Xu F, Wang L (2019) A VTA GABAergic neural circuit mediates visually evoked innate defensive responses. Neuron 103:473–488.e6. 10.1016/j.neuron.2019.05.027 31202540

[B45] Zimmerberg B, Glick SD, Jerussi TP (1974) Neurochemical correlate of a spatial preference in rats. Science 185:623–625. 10.1126/science.185.4151.623 4858234

[B46] Zingg B, Chou X. l, Zhang Z, gangMesik L, Liang F, Tao HW, Zhang LI (2017) AAV-mediated anterograde transsynaptic tagging: mapping corticocollicular input-defined neural pathways for defense behaviors. Neuron 93:33–47. 10.1016/j.neuron.2016.11.045 27989459 PMC5538794

